# Xinmailong Modulates Platelet Function and Inhibits Thrombus Formation *via* the Platelet αIIbβ3-Mediated Signaling Pathway

**DOI:** 10.3389/fphar.2019.00923

**Published:** 2019-08-23

**Authors:** Huawei Wang, Yujia Ye, Wen Wan, Luqiao Wang, Ruijie Li, Longjun Li, Lihong Yang, Lai Yang, Yajuan Gu, Ling Dong, Zhaohui Meng

**Affiliations:** Laboratory of Molecular Cardiology, Department of Cardiology, The First Affiliated Hospital of Kunming Medical University, Kunming, China

**Keywords:** Xinmailong, platelet, thrombosis, α II bβ3, traditional medicine

## Abstract

**Background:** Xinmailong (XML), a bioactive composite extracted from *Periplaneta americana*, has been widely used to treat cardiovascular diseases such as congestive heart failure. However, it is unclear whether XML has antiplatelet and antithrombotic effects.

**Methods:** The effects of XML on agonist-induced platelet aggregation, adhesion and spreading, granule secretion, integrin α II bβ3 activation, and thrombus formation were evaluated. Phosphorylation of Syk, PLCγ2, Akt, GSK3β, and MAPK signaling molecules was also studied on agonist-induced platelets. In addition, the antithrombotic effects of XML were observed *in vivo* using an acute pulmonary thrombosis mouse model.

**Results:** XML dose-dependently inhibited *in vitro* platelet aggregation and granule secretion induced by thrombin, collagen, and arachidonic acid (AA). XML also greatly reduced platelet adhesion and spreading on both collagen- and fibrinogen-coated surfaces. Biochemical analysis revealed that XML inhibited thrombin-, collagen-, and AA-induced phosphorylation of Syk, PLCγ2, Akt, GSK3β, and MAPK. Additionally, XML significantly inhibited *in vivo* thrombus formation in a collagen–epinephrine-induced acute pulmonary thrombosis mouse model.

**Conclusions and General Significance:** Here, we provide the first report showing that XML inhibits platelet function and that it possesses antithrombotic activity. This suggests that XML could be a potential therapeutic candidate to prevent or treat platelet-related cardiovascular diseases.

## Introduction

Thrombotic diseases, such as stroke, myocardial infarction, and arteriosclerosis have become the primary cause of death in humans, and platelets play crucial roles both in physiological hemostasis and pathological thrombosis (Michelson, 2010). Upon vessel wall damage, platelets are exposed to subendothelial extracellular matrix proteins (ECM) such as collagen, which quickly trigger platelet activation and adhesion (Varga-Szabo et al., 2008). Platelets aggregate with each other and release pro-activator substances, such as adenosine diphosphate (ADP), thromboxane A2 (TxA2), and thrombin. These substances further amplify platelet activation and recruit more platelets to form hemostatic thrombi (Watson et al., 2001). Finally, these events activate integrin α II bβ3, allowing platelets to combine with fibrinogen to mediate adhesion and aggregation (Shattil and Newman, 2004). Integrin α II bβ3 maintains an inactive conformation on the surface of resting platelets. Cytoplasmic signals cause it to adopt an active and high-affinity conformation in a process known as “inside-out” signal transduction, which occurs *via* G protein–coupled or tyrosine kinase–linked pathways. Activated integrin α II bβ3 can bind soluble ligands such as fibrinogen and von Willebrand factor (vWF) and subsequently mediate “outside-in” integrin signaling, which leads to integrin clustering, changes in platelet morphology, and postoccupancy events (Mehrbod et al., 2013). These signaling events are responsible for stimulating platelet adhesion and thrombus growth *via* influencing processes such as clot retraction, platelet aggregation, and platelet spreading (Shattil, 1999). The FcγR II A ITAM (immunoreceptor tyrosine-based activation motif)/Syk/PLCγ2 and the PI3K/Akt/GSK3β signaling pathways play a major role in triggering integrin α II bβ3 “outside-in” signaling (Zhi et al., 2013). ERK1/2, JNK1/2, and p38 are also involved in platelet signal transduction (Adam et al., 2008).

A series of antiplatelet drugs have been used clinically in the treatment of atherothrombotic diseases, such as aspirin and clopidogrel. However, these drugs have some side effects, including gastric bleeding and ulcer development. Thus, a special focus has been placed on natural compounds present in medicinal or dietary plants, especially in Chinese traditional herbs, that exhibit antiplatelet activity (Wang et al., 2014). Xinmailong (XML)—a bioactive composite extracted from *Periplaneta americana* (a species of cockroach)—was approved by the China State Food and Drug Administration (CFDA) in 2006 for the treatment of heart failure (HF) (Liu, 2016).

**Figure 1:**
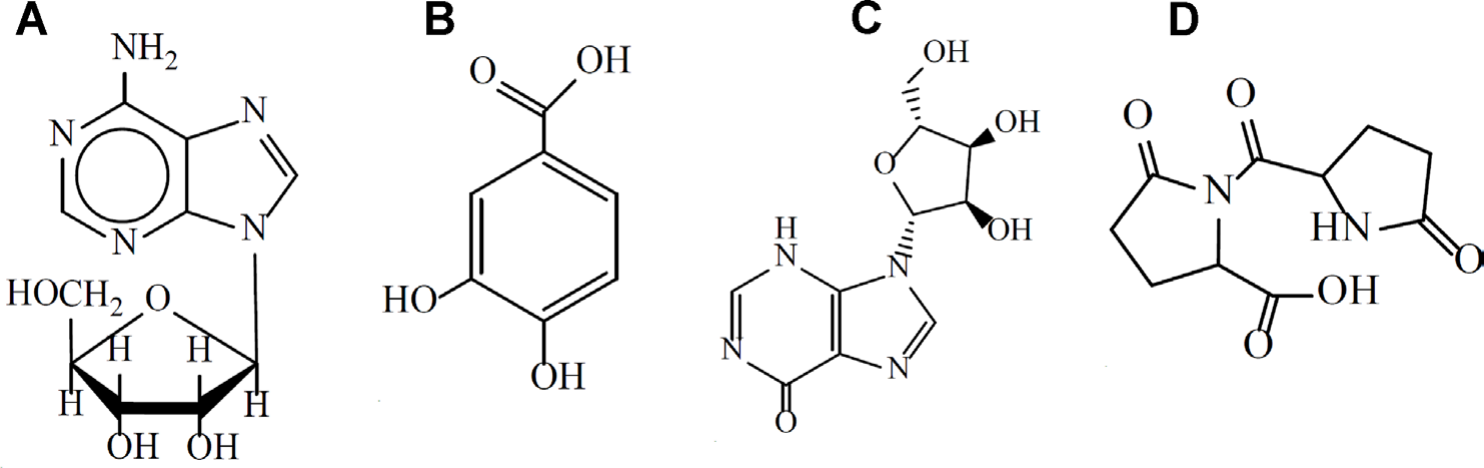
The main active components of XML. **(A)** adenosine, **(B)** protocatechuic acid, **(C)** inosine, **(D)** pyroglutamate dipeptide.

Previously studies have reported the chemical constituents of XML using high-performance liquid chromatography (HPLC) and gas chromatograph–mass spectrometry (GC-MS). 29 compounds have so far been identified, including three of polyhydric alcohols (percentage composition: 38.5%), four of organic acids (percentage composition: 18.8%), a variety of alkaloids (pyrroles, piperidines, piperazines, percentage composition: 6.55%), six of fatty acids and their esters (percentage composition: 5.88%), two of unsaturated lactones, two of amines, two of phenolic compounds, and some microconstituents (such as noradrenaline, ketone compounds, and divinyl sulfide). (Jiao et al., 2011; Jiao et al., 2012) It has been reported that XML contains four main active ingredients: adenosine, inosine, protocatechuic acid, and pyroglutamate dipeptide (**Figures 1A–D**) (Qi et al., 2017). A series of clinical trials performed in HF patients confirmed the effectiveness of XML in improving cardiac function, such as enhanced myocardial contraction and inhibition of ventricular remodeling (Lu et al., 2018). However, no studies have determined whether XML has anti-platelet or anti-thrombotic effects. Therefore, our study focuses on evaluating the effect of XML on platelet activation and thrombosis. Thrombin, collagen, and AA are the most important platelet agonists and can active platelets by inducing signal transduction. In the present study, we used thrombin, collagen, and AA stimulations of platelets to determine if XML differentially regulates platelet function. Here, we provide the first report that XML suppresses thrombin-, collagen-, and AA-induced platelet activation. Moreover, we reveal the potential effects of XML on the thrombin-, collagen-, and AA-stimulated platelet signaling pathway. Based on these studies, we propose that XML inhibits platelet activation through regulation of integrin α II bβ3-mediated signal transduction.

## Materials and Methods

### Chemicals and Reagents

**Figure 2:**
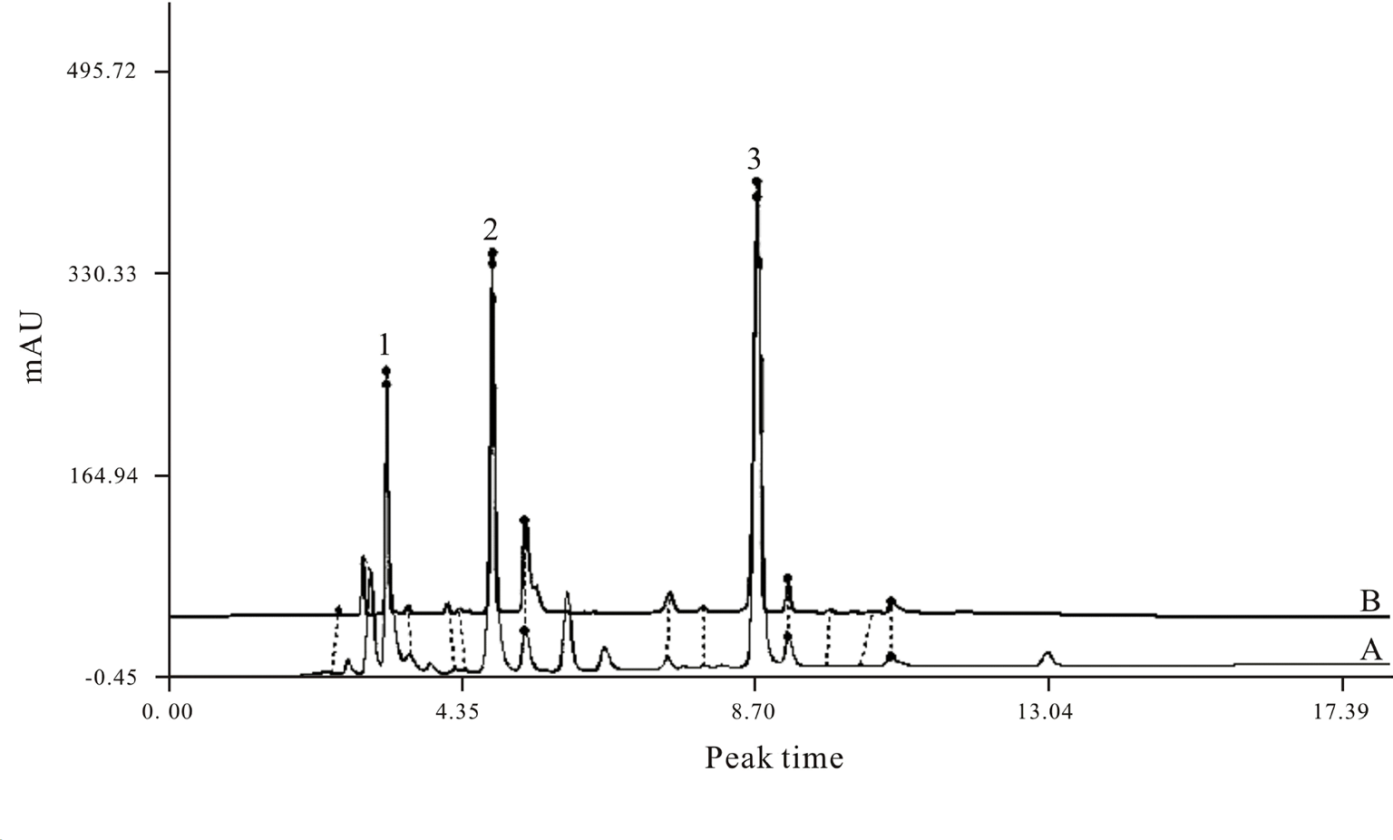
HPLC fingerprint of XML extractum containing three marker compounds. Uracil (C4H4N2O2) (Michelson, 2010), hypoxanthine (C5H4N4O) (Varga-Szabo et al., 2008), inosine (C10H12N4O5) (Watson et al., 2001). **(A)** HPLC fingerprint of standards provided by the CFDA; **(B)** HPLC chromatogram of the XML extractum sample used in the present research. The fingerprint spectrum similarity between the standard and the sample is ≥0.99.

XML used in our research was provided by Yunnan Tengchong Pharmaceutical Corporation (Yunnan, China), in accordance with applicable Good Manufacturing Practice (GMP). We performed HPLC analysis to identify the chemical characteristics of XML samples satisfying the national standards for XML (HPLC≥99%; **Figure 2**). Collagen, thrombin, and arachidonic acid (AA) were purchased from Chrono-Log Corp. (Havertown, PA, USA). Antibodies against total-Syk, phospho-Syk (Tyr525/526), total-PLCγ2, phospho-PLCγ2 (Tyr759), total-Akt, phospho-Akt (Thr308), total-GSK3β, phospho-GSK3β (Ser9), total-Erk1/2, phospho-Erk1/2 (Thr202/Tyr204), total-JNK, phospho-JNK (Thr183/Tyr185), total-p38, and phospho-p38 (Thr180/Tyr182) were obtained from cell signaling (Beverly, MA, USA). CD61 and CD62P (P-selectin) antibodies and PAC-1 antibody were purchased from BD Biosciences (San Jose, CA, USA). ECL Western blotting detection reagent was obtained from Pierce Chemical Co. (Rockford, Illinois, USA). XML was dissolved in saline solution and stored at room temperature until used.

### Platelet Preparation

Human blood was drawn from the cubital vein without stasis into silicon vacutainer tubes containing 1:9 (v/v) 3.8% acid citrate dextrose. Platelet-rich plasma (PRP) was obtained by centrifuging whole anti-coagulant blood for 15 min at 300×*g* after addition of 1 μM prostaglandin E1 (PGE1). After another addition of 1 μM PGE1 and 2.5 mM EDTA, platelets were pelleted by centrifuging the PRP at 300×*g* for 15 min. Samples were then washed twice with Tyrode buffer (137 mM NaCl, 13.8 mM NaHCO_3_, 5.5 mM glucose, 2.5 mM KCl, 20 mM HEPES, 0.36 mM, NaH_2_PO_4_, pH 7.4) and resuspended in 1× Tyrode buffer for final concentration of 3.0 × 10^8^/ml. All of the platelet preparations were conducted at room temperature. CaCl_2_ (1 mM) was added prior to agonist stimulation.

Mouse blood was drawn into a syringe containing 3.8% sodium citrate 1:9 (v/v) from the posterior vena cava of anesthetized Sprague-Dawley rats. Platelets were obtained using the protocol described above.

### Platelet Aggregation and ATP Release Assay

Platelet aggregation and ATP release were measured on a light transmission aggregometer (Chrono-Log, Havertown, PA, USA) as described previously (Ming et al., 2011). The PRP or washed platelets (250 μl, 3.0 × 10^8^/ml) were preincubated with either XML (0.1, 1, 10 mg/ml) or vehicle at 37°C for 10 min and then stimulated with different agonists, while stirring for 6 min at 37°C. Platelet aggregation and secretion (measuring the release of ATP) were recorded. Quantification was obtained by the aggregation and release tracings.

### P-Selectin and PAC-1 Expressions by Flow Cytometric

Washed platelets (3.0 × 10^8^/ml) were pretreated with XML (0.1 mg/ml, 1 mg/ml, 10 mg/ml), or vehicle for 10 min at 37°C and then incubated with collage (5 μg/ml), thrombin (0.1 U/ml), or AA (1 mM) for 5 min at 37°C. The bindings of PerCP-conjugated anti-CD61 antibodies, PE-conjugated anti-CD62P antibodies, and Alexa Fluor–labeled PAC-1 to human platelets (5 × 10^7^/ ml) incubated in the dark at room temperature for 20 min were performed and analyzed with a BD Biosciences flow cytometer (San Jose, CA, USA).

### Platelet Adhesion and Spreading Assays

Twenty four well glass tissue-culture slides were coated with collagen (5 μg/ml) or fibrinogen (25 μg/ml) overnight at 4°C. The slides were washed twice with phosphate buffer saline (PBS) and then, using 1% albumin from bovine serum (BSA), were blocked for 60 min at room temperature. Washed platelets (2 × 10^7^/ml) pretreated with different concentrations of XML (1 and 10 mg/ml) or vehicle were allowed to adhere and spread on glass cover slips coated with collagen or fibrinogen for 45 min at 37°C. Non-adherent platelets were removed by PBS, and adherent platelets were fixed with 2% paraformaldehyde, permeabilized with 0.1% triton X-100, and stained with phalloidin-TRITC (1 μg/ml). Adherent platelets were viewed and photographed using a fluorescence microscope (Olympus, Japan). ImageJ software was used to analyze the number of platelets and the spreading area of individual platelets.

### Clot Retraction Assessment

Platelet clotting was initiated by adding 10 μl of thrombin (10 U/ml) to 500 μl of platelets (2 × 10^7^/ml) in the presence of 400 μg/ml fibrinogen and 1 mM CaCl_2_. The reaction was allowed to proceed for 30 min at 37°C, and photographic images of the clot retraction were recorded.

### Western Blots

Washed platelets were preincubated with vehicle or XML (10 mg/ml) for 10 min. After platelet stimulation with or without agonist for 2 min, reactions were terminated by the addition of an equal volume of 2× lysis buffer (300 mM NaCl, 0.2 mM MgCl_2_, 20 mM EGTA, 30 mM HEPES, 2% Triton X-100, 2× protease, 2× phosphatase inhibitor cocktails). Proteins were electrophoresed on a 10% SDS-PAGE gel and transferred to polyvinylidene difluoride (PVDF) membranes by semi-dry Western blotting. PVDF membranes were blocked using 5% (w/v) BSA, then incubated with the indicated antibodies overnight at 4°C. Membranes were washed with TBST, then exposed to the appropriate secondary antibodies for 1 h at room temperature and detected with chemical luminescence image system.

### Tail-Bleeding Assay

Male mice were divided into the following groups: vehicle, 20 mg/kg XML, or 40 mg/kg XML. Then, the mice were anesthetized with chloral hydrate by intraperitoneal injection. Once anesthetized, vehicle or XML (20 mg/kg, 40 mg/kg) solution was injected *via* the tail vein. After 30 min, tail-bleeding time was measured as previously described (Endale et al., 2012). The distal 5 mm of the tail was transected and immediately immersed into 0.9% saline solution at 37°C. The time between the start of transection to bleeding cessation was recorded as the bleeding time.

### Coagulation Assessment

Rats were segregated into the following groups: vehicle, 20 mg/kg XML, or 40 mg/kg XML. Then, the rats were anesthetized with chloral hydrate by intraperitoneal injection. Once anesthetized, vehicle or XML (20 and 40 mg/kg) solution was injected *via* the tail vein. After 30 min, blood was drawn from the inferior vena cava without stasis into silicon vacutainer tubes containing 1:9 (v/v) 3.8% acid citrate dextrose. Prothrombin time (PT) and activated partial thromboplastin time were detected using an automated coagulation analyzer.

### Collagen–Epinephrine-Induced Acute Pulmonary Thrombosis in Rats

To investigate the effect of XML on thrombosis *in vivo*, the collagen–epinephrine-induced acute pulmonary thrombosis mouse model was adapted. A total of 50 rats were equally divided into five groups as control, vehicle, 50 mg/kg aspirin, 10 mg/kg XML, and 20 mg/kg XML, which were injected *via* the tail vein saline solution, aspirin (50 mg/kg), or different concentration of XML (10 mg/kg, 20 mg/kg), respectively. 30 minutes later, all groups except for control were injected with a mixture of collagen (1.0 mg/kg) and epinephrine (0.1 mg/kg) in the tail vein to induce thrombus formation. The survival time in each group was recorded, and lung sections were visualized using H&E staining.

### Statistical Analysis

All data were analyzed using the Statistical Package for Social Sciences (SPSS, version 20.0) and expressed as the mean ± SEM (standard error of mean). Data were analyzed using one-way ANOVA. Student Newman Keuls (SNK) test was used to determine whether the differences between groups were significant. *P* values less than 0.05 were considered statistically significant.

## Results

### XML Inhibits Human Platelet Aggregation Induced by Various Agonists

**Figure 3:**
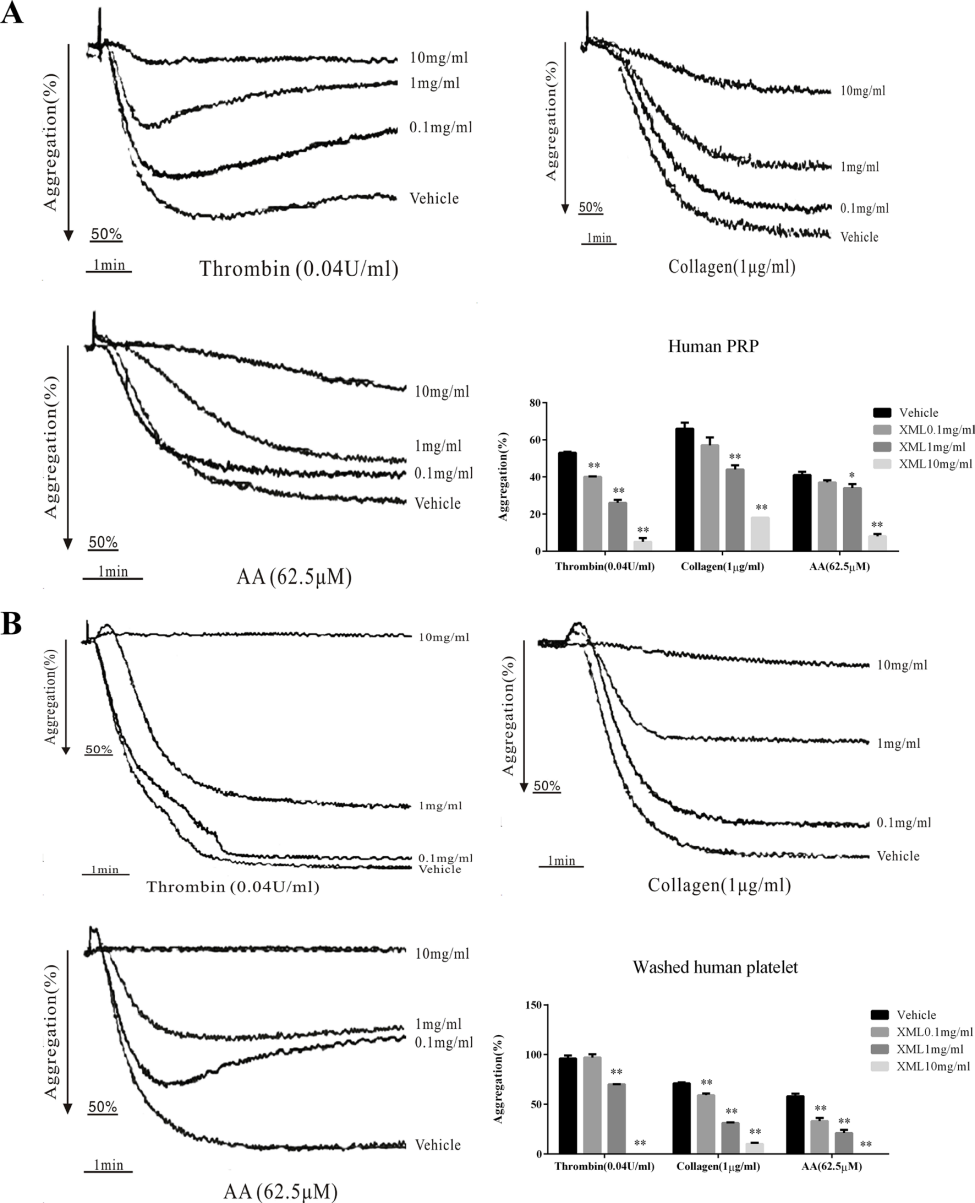
Inhibitory effect of XML on agonist-induced platelet aggregation in human PRP and washed platelets. **(A)** PRP (2.0 × 10^8^/ml) and **(B)** washed platelets (2.0 × 10^8^/ml) were pre-incubated with XML (0.1 mg/ml, 1 mg/ml, and 10 mg/ml) or vehicle prior to the addition of thrombin (0.04 U/ml), collagen (1 μg/ml), or AA (62.5 μM) to trigger platelet aggregation. Quantification of aggregation (%) is also shown in the lower panel. Data are expressed as the mean ± SEM of three experiments. *P < 0.05, **P < 0.01 *vs*. vehicle.

XML (0.1 mg/ml, 1 mg/ml, 10 mg/ml) exhibited a dose-dependent inhibitory effect on platelet aggregation stimulated by collagen (1 μg/ml), thrombin (0.04 U/ml), and AA (62.5 μM) in human PRP (**Figure 3A**); 1 mg/ml XML significantly decreased collagen-induced platelet aggregation to 44.0 ± 2.3% from 66.0 ± 3.3% (n = 4; *P* < 0.01), thrombin-induced platelet aggregation to 26.0 ± 1.7% from 53.0 ± 0.6% (n = 4; *P* < 0.01), and AA-induced platelet aggregation to 34.0 ± 2.2% from 41.0 ± 1.9% (n = 4; *P* < 0.05). 10 mg/ml XML almost fully abolished thrombin-induced platelet aggregation (5.0 ± 2.0%) and AA-induced platelet aggregation (8.0 ± 1.3%) and decreased collagen-induced platelet aggregation to 18.0 ± 0%. Moreover, in washed human platelets, XML (0.1, 1, 10 mg/ml) also exhibited a dose-dependent inhibitory effect on platelet aggregation stimulated by collagen (1 μg/ml), thrombin (0.04 U/ml), and AA (62.5 μM) (**Figure 3B**).

**Figure 4:**
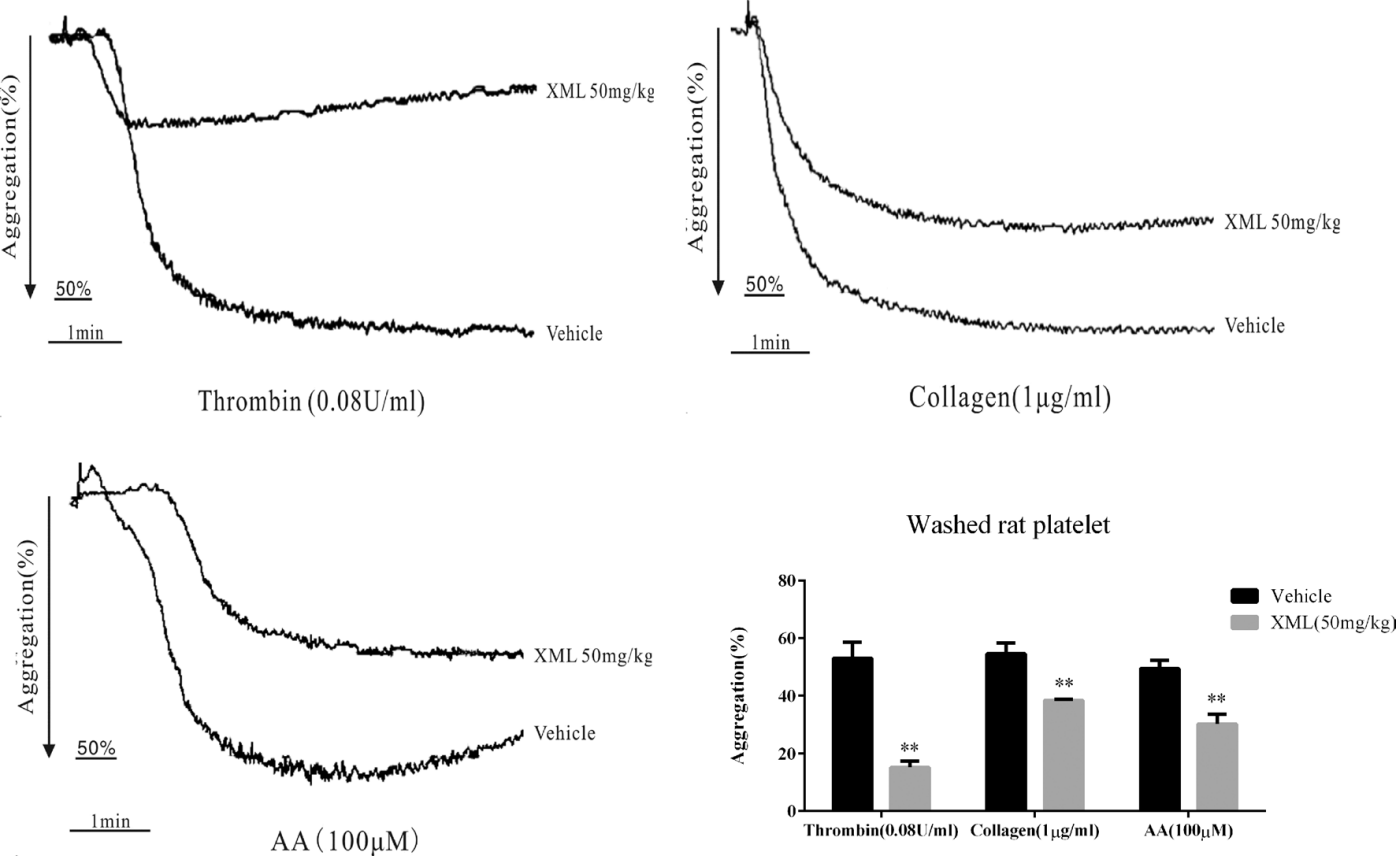
XML affects platelet activity *in vivo* in rats. Rats were pre-treated with vehicle, 50 mg/kg XML by tail vein injection for 30 min. Then, washed platelets (3.0 × 10^8^/ml) were treated with thrombin (0.08 U/ml), collagen (1 μg/ml), or AA (100 μM) to trigger platelet aggregation. Quantification of aggregation (%) is shown in the lower panel. Data are expressed as the mean ± SEM of 10 experiments. **P <0.01 *vs*. vehicle.

To further investigate the effect of XML on platelets *in vivo*, rats were treated with vehicle or 50 mg/kg XML. As shown in **Figure 4**, XML inhibited platelet aggregation induced by collagen (1 μg/ml), thrombin (0.08 U/ml), and AA (100 μM) (54.6 ± 3.8 to 38.4 ± 0.4%, *P* < 0.01, 53.0 ± 5.5 to 15.2 ± 2.2%, *P* < 0.01, and 49.4 ± 2.9 to 30.2 ± 3.5%, respectively, *P* < 0.01, n = 10).

### XML Inhibits Agonist-Induced Platelet Granule Secretion and Integrin α II bβ3 Activation

**Figure 5:**
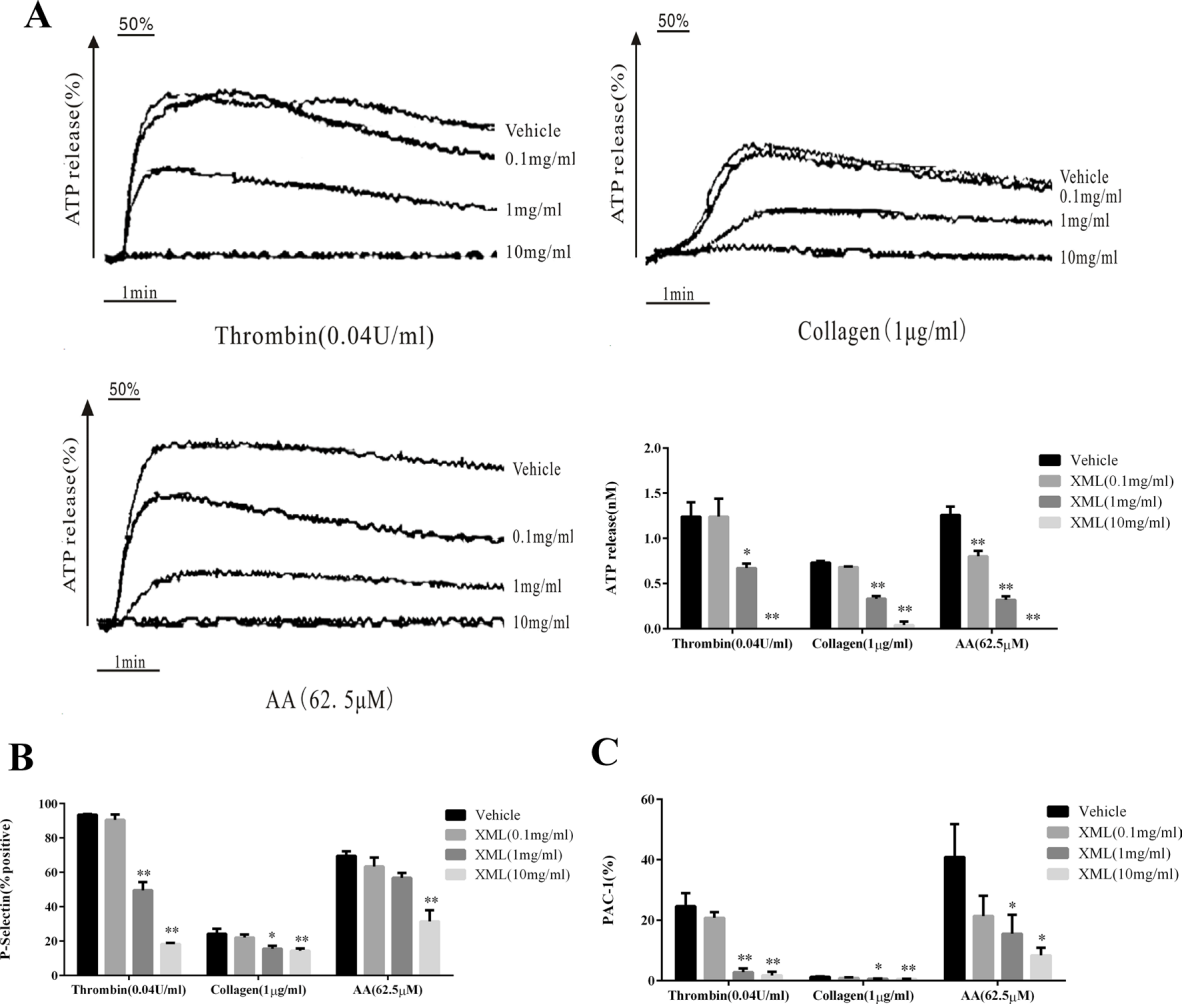
Inhibitory effect of XML on agonist-induced ATP-release, P-, and PAC-1 expressions in washed human platelets. **(A)** Washed platelets (5.0 × 10^7^/ml) were pre-treated with XML (0.1, 1, and 10 mg/ml) or vehicle before the addition of thrombin (0.04 U/ml), collagen (1 μg/ml), or AA (62.5 μM) in the ATP-release reaction experiment. Quantification of ATP release (nM) is shown in the right panel. **(B, C)** Washed human platelets (5.0 × 10^7^/ml) were pre-incubated with XML (0.1, 1, and 10 mg/ml) at 37°C for 10 min in the presence of PE-conjugated P-selectin and Alexa Fluor–conjugated PAC-1 antibodies, stimulated with thrombin (0.04 U/ml), collagen (1 μg/ml), or AA (62.5 μM), and incubated for a further 5 min. P-selectin expression (%) and PAC-1 expression (%) were determined by flow cytometry. Quantification of P-selectin expression (%) **(B)** and PAC-1 expression (%) **(C)** are shown in the lower panel. The results are expressed as mean ± SEM of three experiments. *P <0.05, **P <0.01 *vs*. vehicle.

To investigate the effects of XML on platelet secretion properties, we treated washed human platelets preincubated with various concentrations of XML (0.1, 1, 10 mg/ml) to thrombin (0.04 U/ml), collagen (1 μg/ml), and AA (62.5 μM). Our data show that platelet dense granule secretion was significantly inhibited by XML (**Figure 5A**). Moreover, we found that P-selectin expression induced was also significantly reduced by XML (0.1, 1, 10 mg/ml). 10 mg/ml XML significantly inhibited all thrombin-, collagen-, and AA-induced surface expression of P-selectin (18.2 ± 0.7%, 14.4 ± 1.3%, 31.4 ± 6.5%, respectively, **Figure 5B**). PAC-1 binding induced by thrombin, collagen, and AA was also blocked by XML (0.1, 1, 10 mg/ml). The level of PAC-1 binding to thrombin-, collagen-, and AA-stimulated platelets was reduced from 24.6 ± 4.3 to 1.7 ± 1.2%, from 1.2 ± 0.2%, to 0.5 ± 0.1%, and from 40.9 ± 10.9 to 8.4 ± 2.5%, respectively) (**Figure 5C**).

### XML Inhibits Collagen-Induced Platelet Adhesion

**Figure 6:**
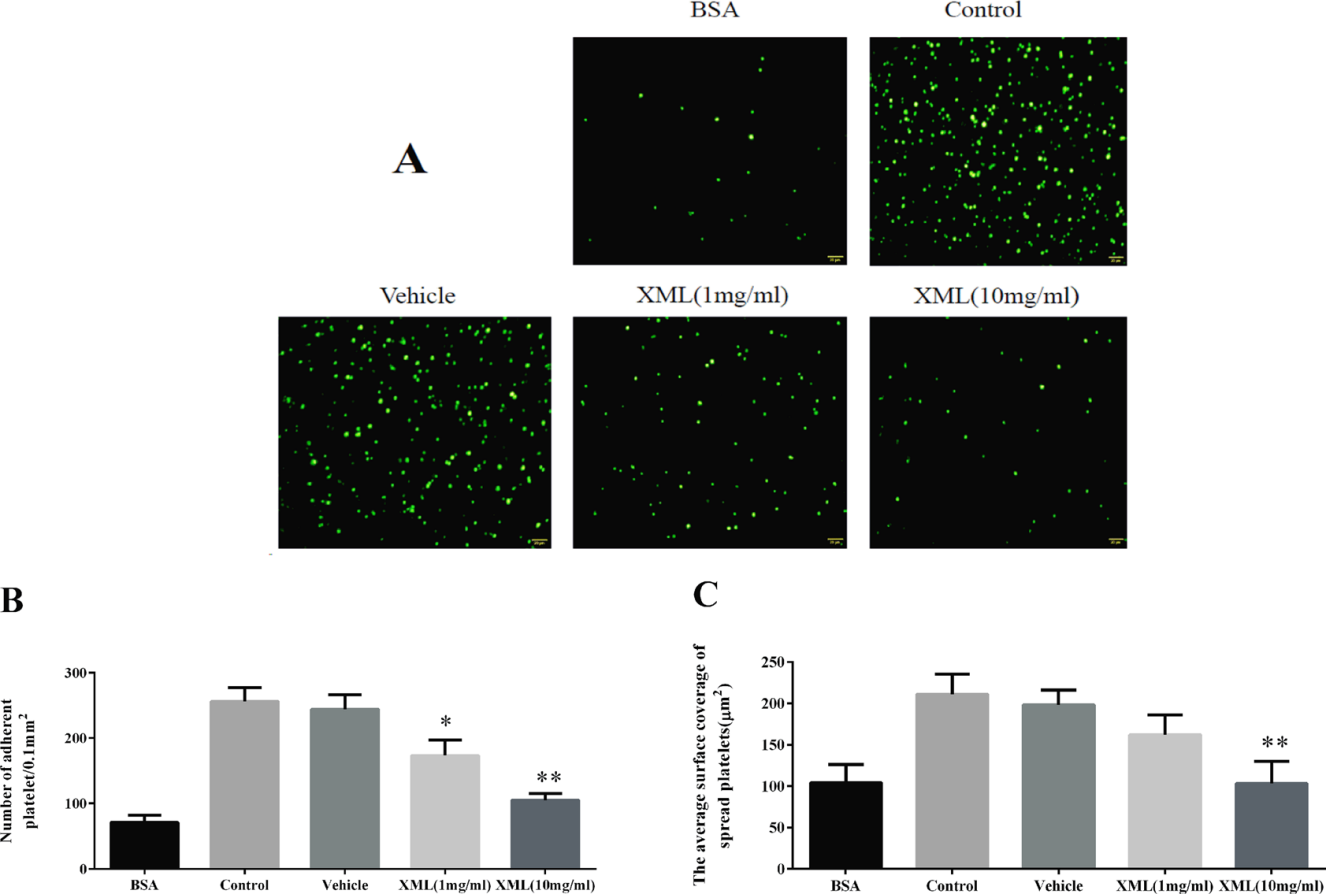
XML inhibits platelet adhesion on collagen-coated surfaces. **(A)** Human washed platelets (2.0 × 10^7^/ml) were pre-treated with vehicle or XML (1 mg/ml, 10 mg/ml) for 10 min at 37°C, then allowed to adhere on collagen-coated slides. We selected representative images from three independent experiments. **(B)** The number of adherent platelets and **(C)** the average surface area of individual platelets was analyzed. (Mean ± SEM, n = 3, *P <0.05, **P <0.01 *vs*. vehicle).

Next, we investigated whether XML plays a role in collagen-induced platelet adhesion and spreading. On immobilized collagen surfaces, XML (1 mg/ml, 10 mg/ml) inhibited platelet adhesion in a dose-dependent fashion (**Figure 6**). Compared to control, 1 mg/ml XML and 10 mg/ml XML inhibited platelet adhesion from 256 ± 21 platelets/0.01 to 173 ± 24 platelets/0.01 and 105 ± 10 platelets/0.01 mm^2^, respectively. Furthermore, 1 mg/ml XML and 10 mg/ml XML decreased average surface coverage of a single spreading platelet from 211 ± 24 to 162 ± 24 μm^2^ and 103 ± 27 μm^2^, respectively.

### XML Inhibits Activation of Platelet Signaling Pathways by Thrombin, Collagen, and AA

**Figure 7:**
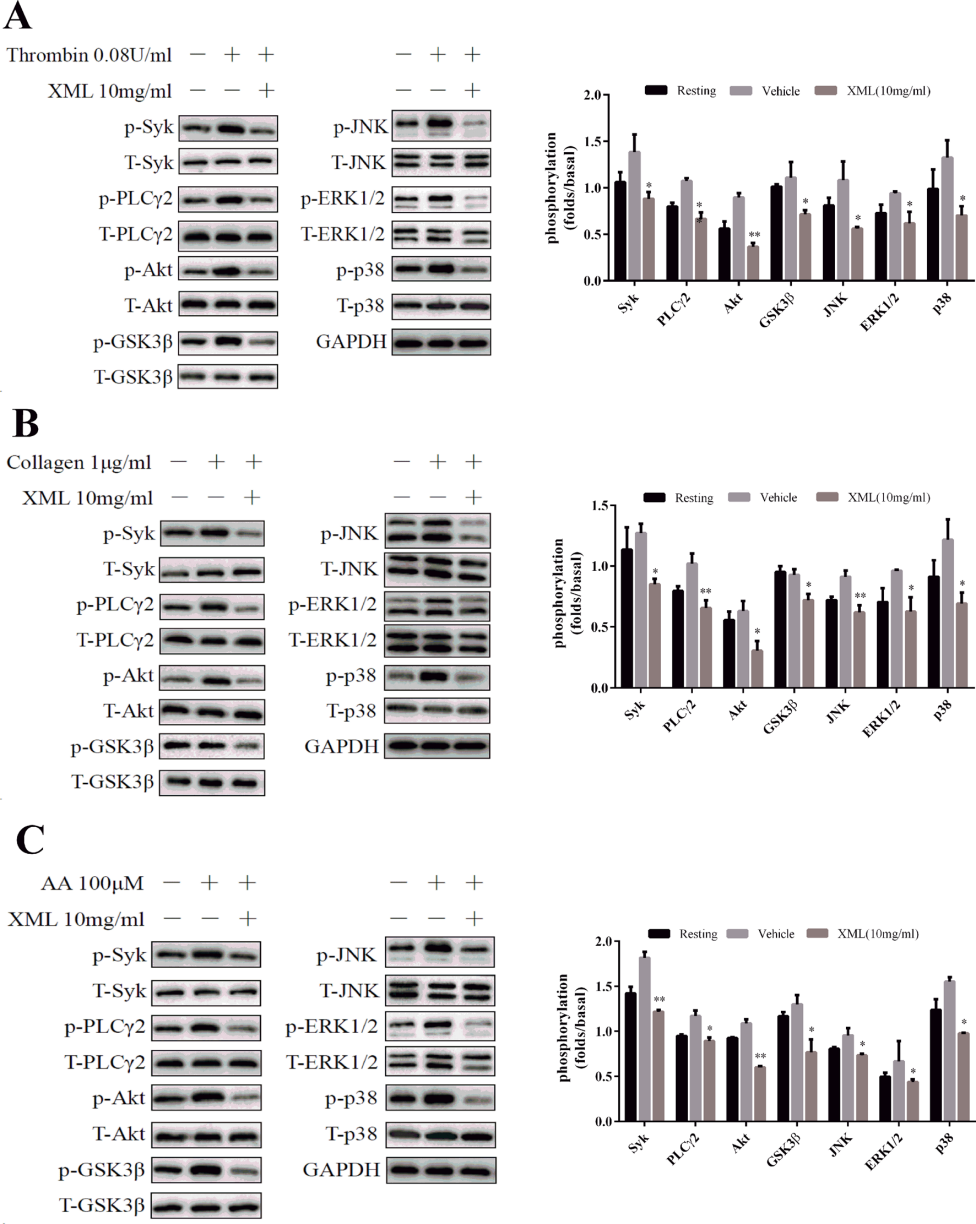
Effect of XML on agonist-induced signal transduction in washed human platelets. Washed platelets (5.0 × 10^8^/ml) were stimulated by **(A)** 0.08 U/ ml thrombin, **(B)** 1 μg/ml collagen, and **(C)** 100 μM AA for 120 s and subsequently lysed with cell lysis buffer. The levels of the phosphorylation of Syk, PLCγ2, Akt, GSK3β, JNK, ERK1/2, and p-38 were detected with the relevant antibodies. Total protein and GAPDH were used as loading controls. The band density was calculated with ImageJ. Values are expressed as the mean ± SEM (n = 3). *P <0.05, **P <0.01 *vs*. vehicle.

Several signaling pathways, including Syk-PLCγ2-MAPK and PI3K-Akt-GSK3β, were evaluated. We found that agonist-induced Syk and PLCγ2 phosphorylation were significantly inhibited by XML (10 mg/ml) treatment (**Figure 7**). Next, we examined phosphorylation levels of mitogen-activated protein kinase (MAPK) signaling pathways. We found that thrombin, collagen, and AA stimulations led to the activation of MAPKs (JNK, ERK1/2, and p38) in platelets, and that XML (10 mg/ml) blocked this activation (**Figure 7**). These results suggest that XML may be a negative regulator of Syk-PLCγ2-MAPK signaling.

Furthermore, Akt phosphorylation was detected after platelets were stimulated by thrombin, collagen, and AA. As shown in **Figure 7**, XML (10 mg/ml) inhibited this process. Similarly, thrombin-, collagen-, and AA-induced phosphorylation of GSK3β, a substrate of Akt, was attenuated by XML (10 mg/ml) (**Figure 7**). These findings demonstrate that XML may function as a negative regulator of PI3K-Akt-GSK3β signaling pathways.

**Figure 8:**
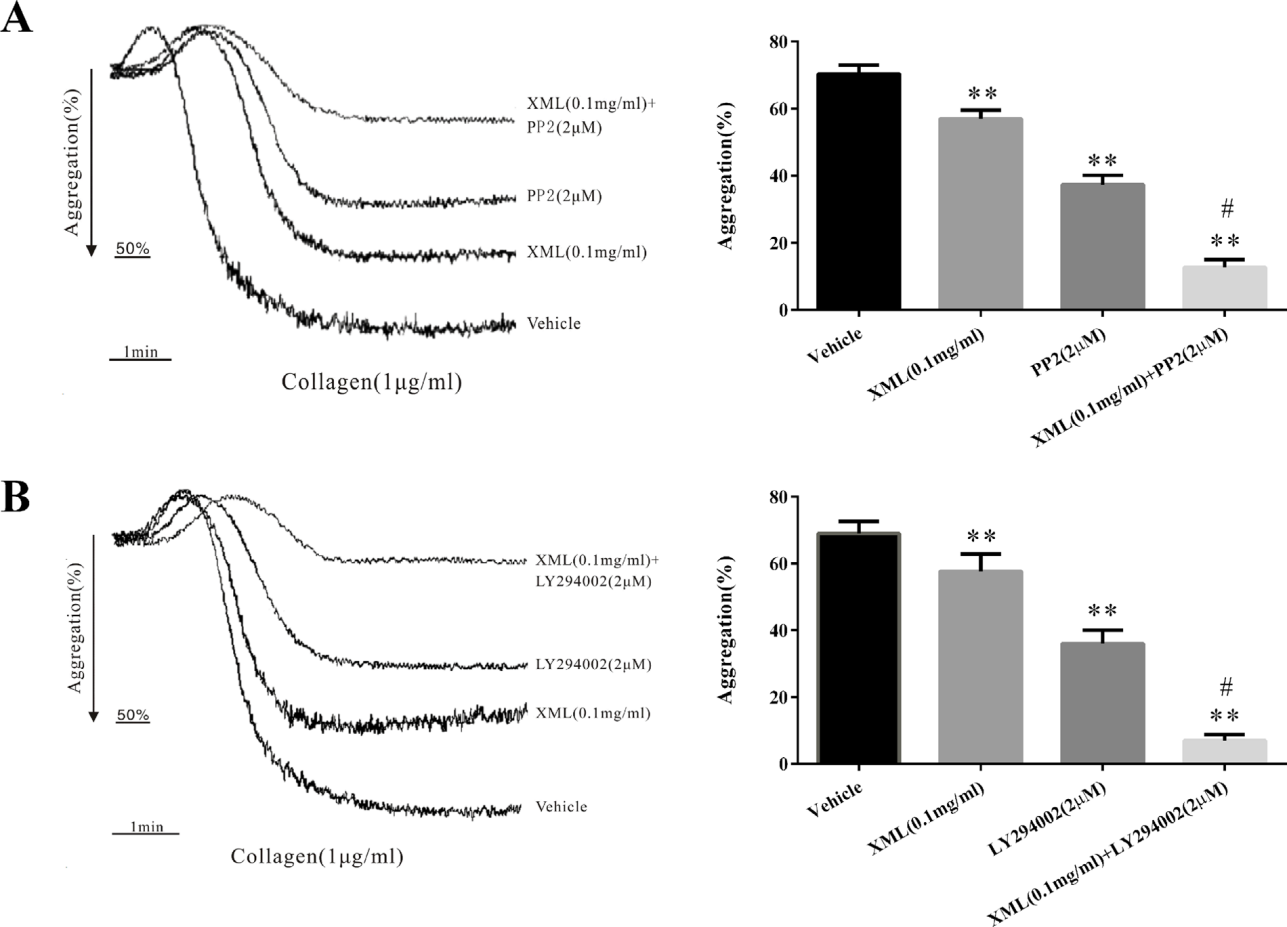
XML negatively regulated signaling. **(A)** Platelets were pretreated with either XML (0.1 mg/ml), PP2 (SFK inhibitor, 2 μM), or XML (0.1 mg/ml) and PP2 (2 μM) before collagen stimulation. **(B)** Platelets were pretreated with either XML (0.1 mg/ml), LY294002 (PI3K inhibitor, 2 μM), or XML (0.1 mg/ml) and LY294002 (2 μM) before collagen stimulation. Bar graphs show mean ± SEM of three experiments. *P <0.05, **P <0.01 *vs*. vehicle. ^#^ *P* <0.01 *vs*. PP2 or LY294002 group.

Finally, platelets were pretreated with XML (0.1 mg/ml), PP2 (SFK inhibitor, 2 μM), or LY294002 (PI3K inhibitor, 2 μM), before collagen stimulation. Additionally, platelets were treated with XML (0.1 mg/ml) and PP2 (2 μM) or with XML (0.1 mg/ml) and LY294002 (2 μM) before collagen (1 μg/ml) stimulation. As shown in **Figure 8A**, combined XML (0.1 mg/ml) and PP2 (2 μM) treatment exhibited inhibitory effects on collagen-induced platelet aggregation. We obtained the similar results when treating platelets with XML (0.1 mg/ml) and LY294002 (2 μM) (**Figure 8B**). All of the above observations suggest that XML may act on multiple signaling pathways to suppress platelet function.

### XML Negatively Regulates Integrin α II bβ3-Mediated “Outside-In” Signaling

As platelet aggregation is dependent upon the association of activated integrin α II bβ3 in complex with its soluble ligand, fibrinogen, we further study clot retraction and platelet spreading on fibrinogen-coated surfaces.

**Figure 9:**
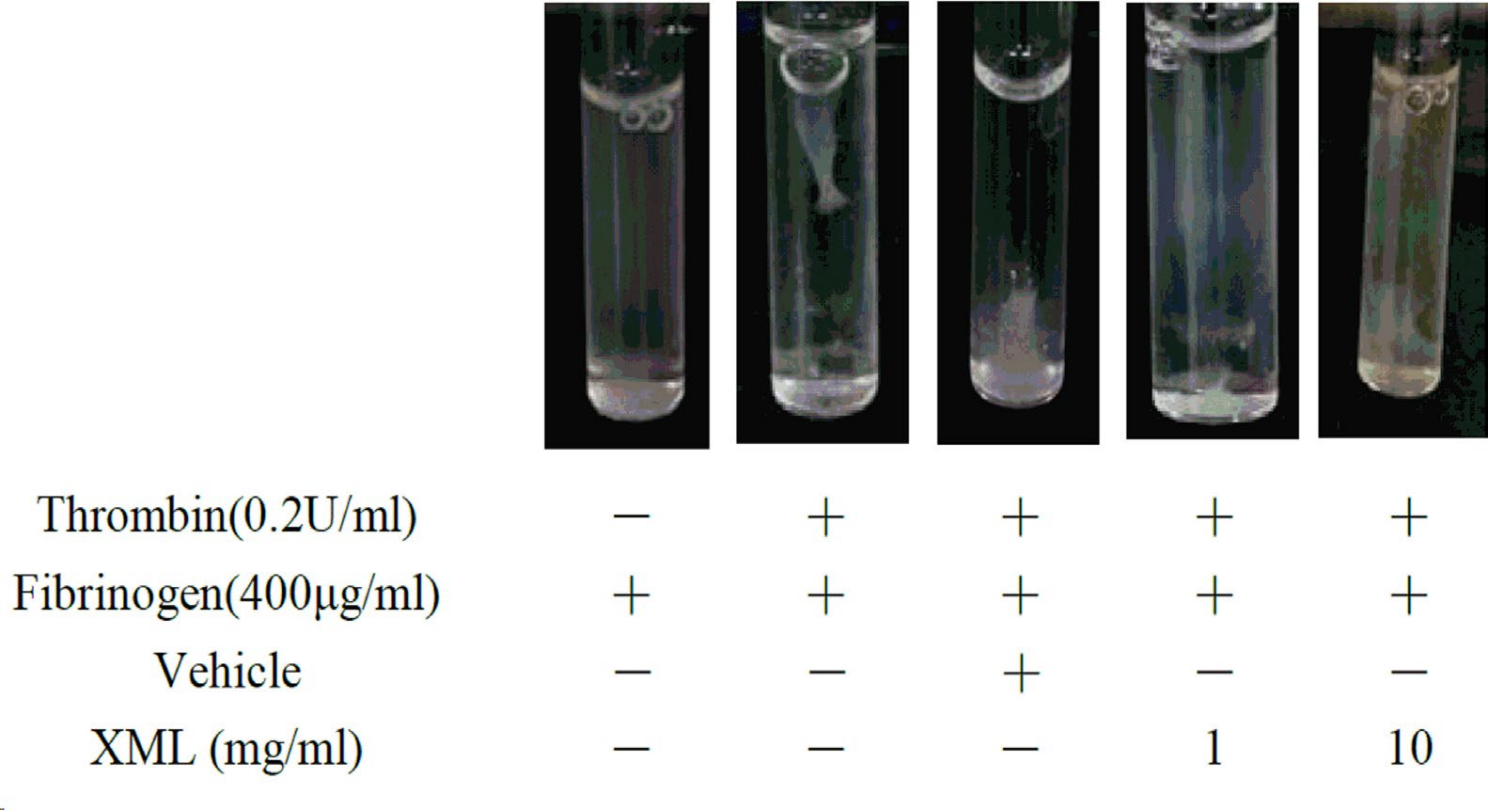
XML inhibits clot retraction. The clot retraction assay was performed, and images were taken 30 min later. Representative results were selected from three independent experiments.

Clot retraction is an integrin α II bβ3-mediated function involving cytoskeletal reorganization. As shown in **Figure 9**, a substantial reduction in clot retraction was observed in the presence of XML-treated (1, 10 mg/ml) platelets compared with the vehicle group.

**Figure 10:**
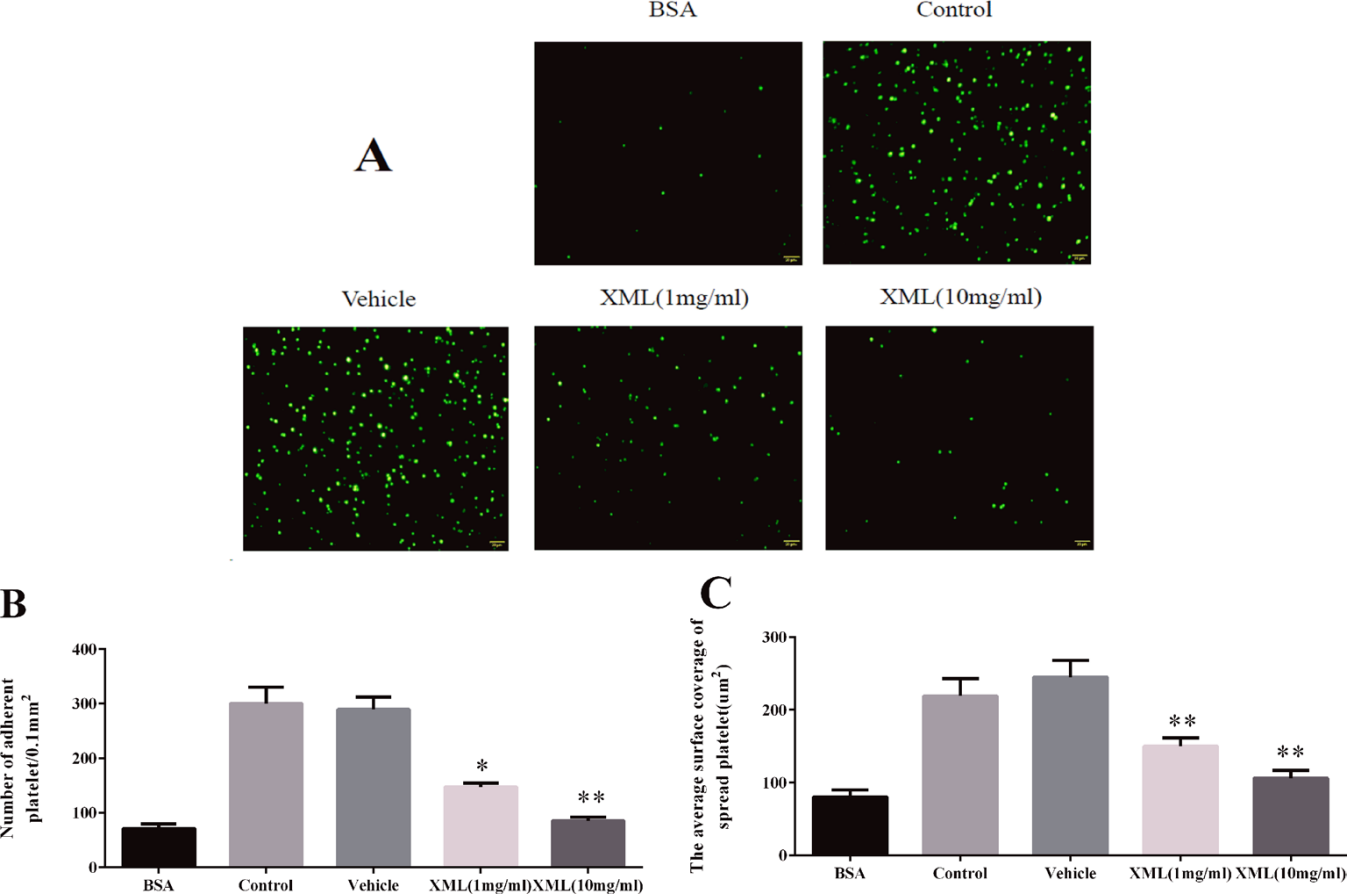
XML inhibits platelet spreading on fibrinogen-coated surfaces. **(A)** Human washed platelets (2.0 × 10^7^/ml) were pre-treated with vehicle or XML (1 mg/ml, 10 mg/ml) for 10 min at 37°C, then allowed to adhere to fibrinogen-coated slides. We selected representative images from three independent experiments. **(B)** The number of adherent platelets and **(C)** the average surface area of individual platelets was analyzed. (mean ± SEM, n = 3, *P <0.05, **P <0.01 *vs*. vehicle).

Next, we examined platelet spreading on fibrinogen-coated surfaces. As shown in **Figure 10**, 1 mg/ml XML- and 10 mg/ml XML-treated platelets stained with TRITC-conjugated phalloidin displayed reduced adhesion and spreading on fibrinogen-coated surfaces compared with vehicle-treated platelets. The number of platelets stuck to immobilized fibrinogen was reduced from 300 ± 30 platelets/0.01 mm^2^ to 147 ± 7 platelets/0.01 mm^2^ (1 mg/ml XML) and 85 ± 7 platelets/0.01 mm^2^ (10 mg/ml XML), and the surface coverage of a single platelet was decreased from 249 ± 24 μm^2^ to 150 ± 11 μm ^2^ (1 mg/ml XML) and 106 ± 11 μm ^2^ (10 mg/ml XML).

### Effect of XML on Coagulation Function

**Figure 11:**
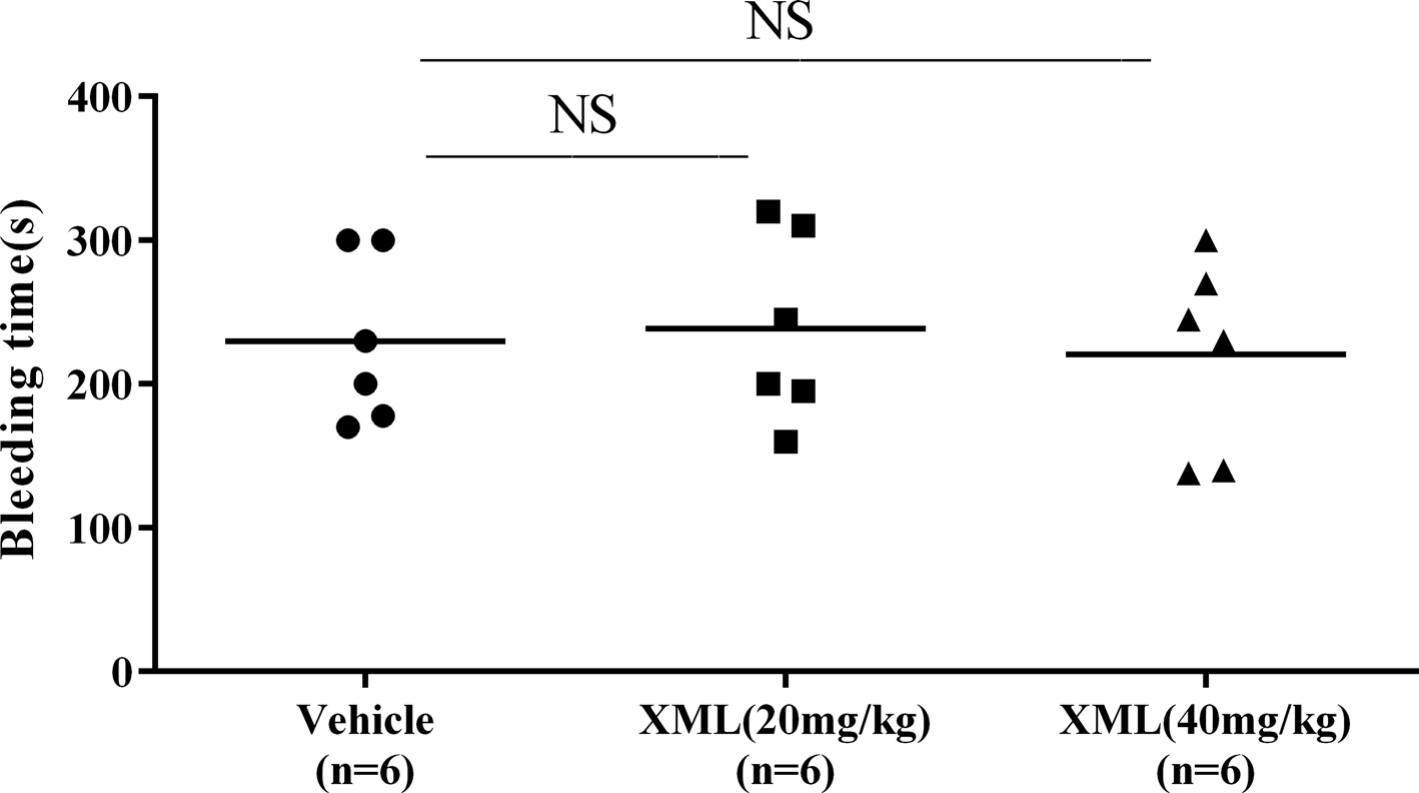
Effect of XML on hemorrhage and hemostasis. The mouse tails were transected to induce bleeding, and the time to bleeding cessation was recorded. Data are reported as means ± SEM (n = 6). N.S., no significant difference *versus* vehicle.

To assess the effect of XML on hemorrhage and hemostasis, tail-bleeding time was measured in mice. Animals were randomly segregated into three groups (vehicle, 20 XML, 40 mg/kg XML). Once anesthetized, vehicle or XML (20 mg/kg, 40 mg/kg) solution was injected *via* the tail vein. 30 min after XML administration, tail-bleeding time was assessed. As shown in **Figure 11**, administration of XML (20, 40 mg/kg) did not extend bleeding time compared to the vehicle group. (*P* > 0.05, n = 6).

**Figure 12:**
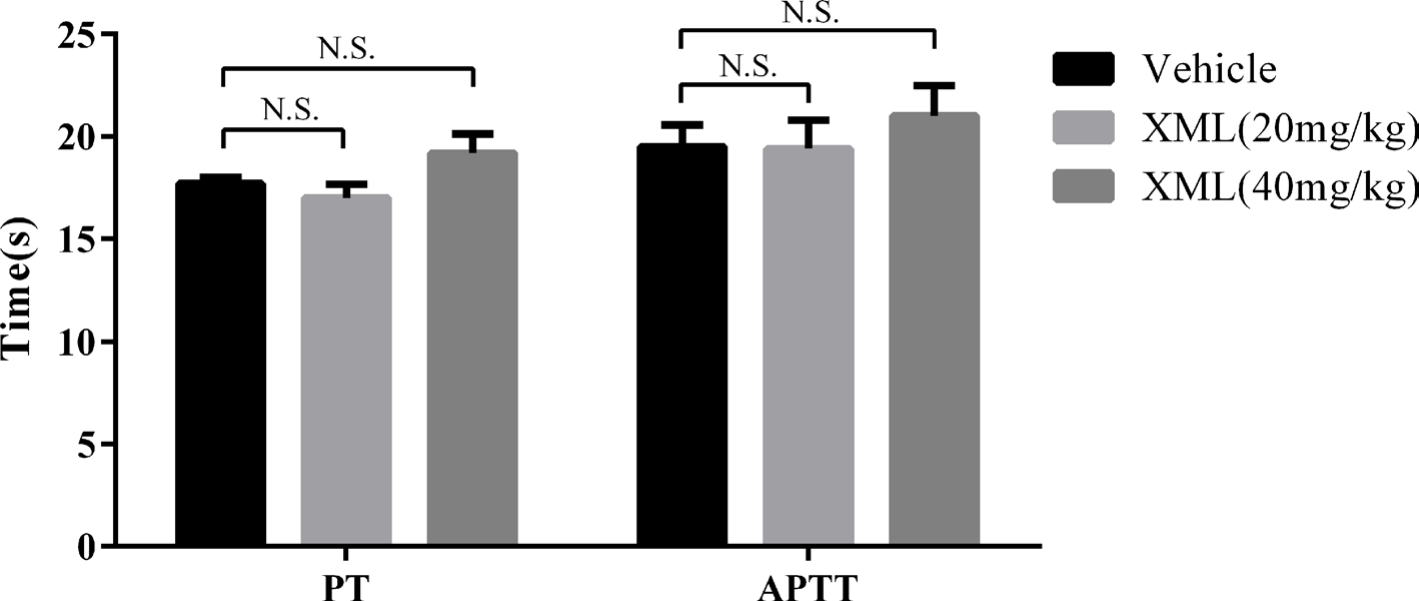
Effect of XML on prothrombin time (PT) and activated partial thromboplastin time (APTT). Results are expressed as means ± SEM (n = 10). N.S., no significant difference *versus* vehicle.

To investigate the effect of XML on coagulation *in vivo*, rats were segregated into vehicle, 20 mg/kg XML, or 40 mg/kg XML groups. Once anesthetized, vehicle or XML solution was injected *via* the tail vein. After 30 min, blood was drawn from the inferior vena cava. As shown in **Figure 12**, we found that neither PT nor activated partial thromboplastin time (APTT) was affected by different doses of XML (20, 40 mg/kg) (*P* > 0.05, n = 10), indicating that XML has no effect on coagulation *in vivo*.

### XML Inhibited Thrombus Formation *In Vivo*

**Figure 13:**
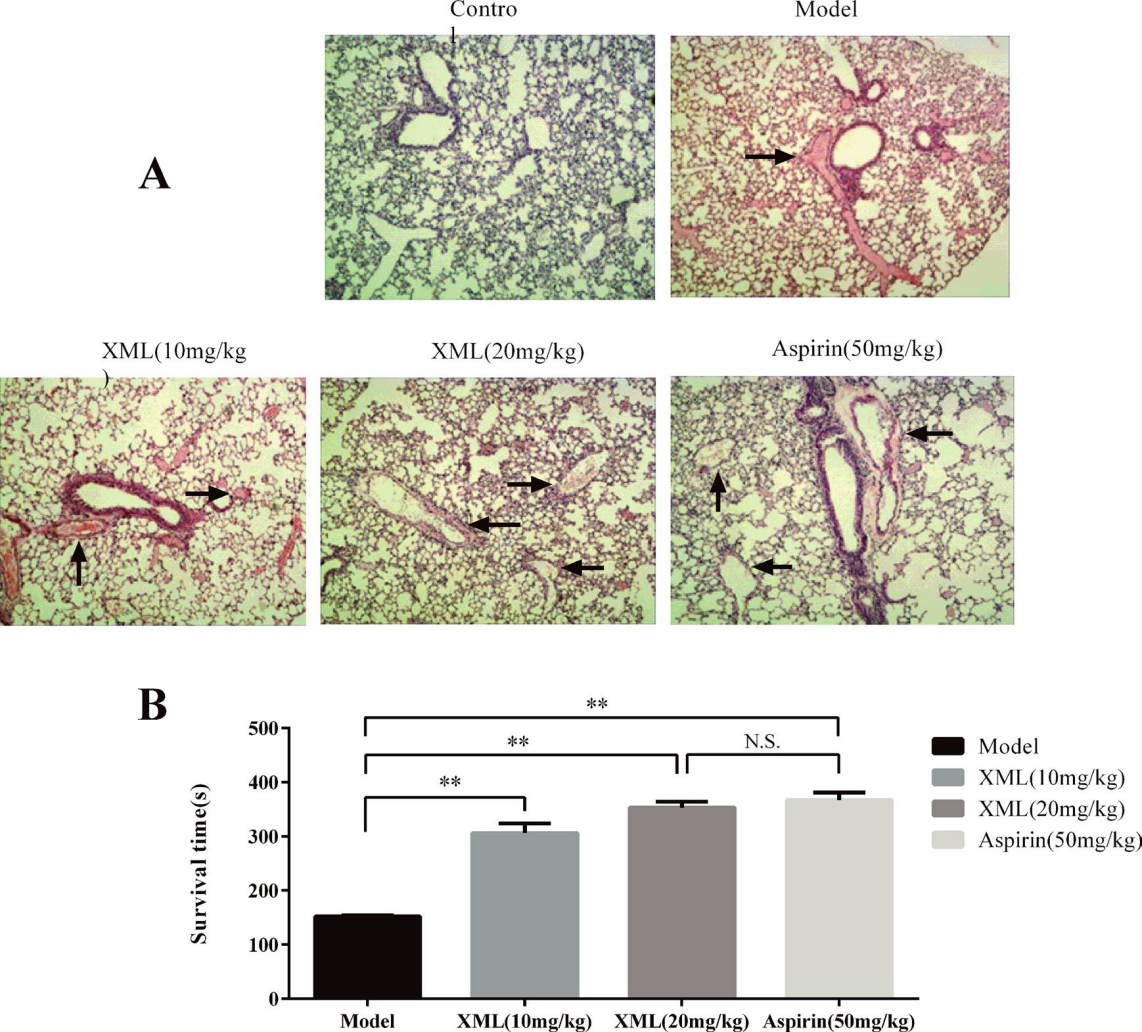
XML- or aspirin-treated mice were protected from lung vascular thrombosis. Mice were pre-treated with 10 mg/kg XML, 20 mg/kg XML, or 50 mg/kg aspirin for 30 min prior to the intravenous injection of collagen (1.0 mg/kg) and epinephrine (0.1 mg/kg). **(A)** Representative sections of H&E-stained lung sections from mice treated as labeled. Magnification: ×200. **(B)** Survival time of collagen-/epinephrine-induced mice injected with XML or aspirin. XML was effective in preventing collagen/epinephrine-induced thromboembolic events (n = 10). **P <0.01 *vs*. model. N.S., means no significant differences *vs*. model.

To explore the effect of XML on thrombosis *in vivo*, we developed an acute pulmonary thrombosis mouse model using collagen–epinephrine-induced as described in the *“Materials and methods”* section. We used vehicle, 10 mg/kg XML, 20 mg/kg XML, and 50 mg/kg aspirin treatment to examine thrombus formation. As shown in **Figure 13A**, platelet thrombi formed throughout the pulmonary vasculature in the vehicle group. However, the lungs of mice injected with either 10 mg/kg XML, 20 mg/kg XML, or 50 mg/kg aspirin showed a substantial reduction in thrombus formation. Importantly, aspirin 50 mg/kg and XML (20 mg/kg, 40 mg/kg) treatment prolonged mice survival time (**Figure 13B**). These data demonstrate that XML may alleviate thrombosis *in vivo*.

## Discussion

This study provides the first report of the antiplatelet activity of XML. Using several approaches, we have defined the effects of XML on platelet function, characterized underlying mechanism, and evaluated its ability to regulate platelet-mediated thrombus formation *in vivo*. This study suggests that XML inhibits agonist-induced platelet activation and integrin α II bβ3-mediated signaling, and that it exerts anti-thrombotic activity *in vivo*. XML inhibited collagen-, thrombin-, and AA-induced platelet aggregations and also inhibited ATP secretion and P-selectin expression. Furthermore, XML reduced platelet adhesion and spreading when immobilized on both collagen- and fibrinogen-coated surfaces and attenuated platelet clot retraction. These results suggest that XML inhibits integrin α II bβ3-mediated signaling. XML impaired agonist-induced phosphorylation of molecules including those involved in the GPVI-Syk-PLCγ2-MAPK and PI3K-Akt-GSK3β signaling pathways. Finally, XML serves to negatively regulate platelet thrombus formation after collagen–epinephrine-induced acute pulmonary thrombosis *in vivo*.

Under physiological conditions, maintenance of platelet quiescence requires a balance between activation and inhibition signaling events. Healthy arterial endothelium is able to provide powerful inhibitory signaling to platelets. However, upon vascular injury, platelets are activated by adhesion to adhesive proteins, such as von Wille-brand factor (vWF) and collagen, or by soluble platelet agonists, such as thrombin, TXA_2_, and ADP. Platelets adhere to collagen and aggregate, initiating early signaling events (Li et al., 2010). Platelet responses to collagen are mediated through glycoprotein VI (GPVI), which initiates Src family kinase (SFK)-based signaling transduction and the activation of the GPVI-Syk-PLCγ2-MAPK and PI3K-Akt-GSK3β signaling pathways (Ragab et al., 2007; Goggs and Poole, 2012). PLCγ2, a downstream molecule of Syk, subsequently induces the formation of the second messengers IP3 and DAG, leading to calcium mobilization, granule secretion, platelet aggregation, and integrin α II bβ3 regulation (Harrison et al., 2018). MAPKs are a downstream target of SFK, and many studies have shown that MAPKs including ERK, JNK, and p38 contribute significantly to platelet activation by various agonists (Chang and Karin, 2001). ERK has been shown to enhance platelet aggregation and secretion, while JNK is thought to decrease integrin α II bβ3 activation and impair granule secretion. p38 is involved in platelet spreading and adhesion (Kuliopulos et al., 2004; Adam et al., 2008; Adam et al., 2010). PI3K signaling contributes to collagen-/GPVI-mediated activation of PLCγ2 by generating PIP3, which induces PLCγ2 translocation to the cell membrane. This is essential for full activation of PLCγ2 (Ragab et al., 2007). Akt, a downstream molecule of PI3K, and its substrate GSK3β play a vital role in platelet activation as shown by several studies (Li et al., 2008; O’Brien et al., 2011). AA can synthesize TXA_2_ in activated platelets through the cyclooxygenase (COX) pathway. Once formed, TXA_2_ diffuses across the membrane and activate other platelets *via* its receptors: TPα and TPβ, which couple to the G_q_ and G_12/13_ proteins. Thrombin stimulates platelet activation mainly *via* G-protein-linked protease-activated receptors (PAR_S_), which couple to the proteins G_12/13_, G_q_, and G_i_ (Davì and Patrono, 2007). Gq protein-coupled receptors stimulated the β_2_ and β_3_ isoforms of phospholipase C (PLC). PLC activation leads to calcium mobilization and activation of protein kinase C (PKC). Gq proteins also stimulate the PI3K/Akt pathway, resulting in changes in platelet morphology, degranulation, and integrin-mediated aggregation (Rivera et al., 2009). Thus, thrombin and AA utilize a common signaling pathway that can activate PLCβ/PKC. It is well known that most agonists activate platelets through G-protein-coupled receptors, leading to integrin glycoprotein II b/III a (α II bβ3) activation, which is the final step in platelet activation (Davì and Patrono, 2007; Stegner and Nieswandt, 2011).

In our study, we found that platelet adherence to immobilized collagen-coated surfaces was decreased by XML. Similarly, XML inhibited collagen-, thrombin-, and AA-induced platelet aggregations in dose-dependent fashion.

When platelets are activated, intracellular secretory granules such as α-granules, dense granules, and lysosomes are secreted, which is key to platelet activation and thrombus stabilization. The α-granules are the most abundant and contain many bioactive proteins, such as vWF, fibrinogen, and P-selectin (Diacovo et al., 1996; Sharda and Flaumenhaft, 2018). Thus, we examined the expression of immunofluorescence-labeled P-selectin to identify α-granule secretion. XML reversed collagen-, thrombin-, and AA-induced platelet P-selectin expressions. We detected ATP release to show dense granule secretion (McNicol and Israels, 1999). Consistently, XML significantly reduced collagen-, thrombin-, and AA-induced ATP releases from platelets. These observations imply that XML may exert its antiplatelet effect by suppressing secretory granule release.

In our experimental conditions, XML inhibited collagen-, thrombin-, and AA-induced phosphorylations of molecules involved in the GPVI-Syk-PLCγ2-MAPK and PI3K-Akt-GSK3β signaling pathways, indicating that XML may exert its inhibitory effects on stimulated platelets through a common signaling cascade.

Our study shows that XML significantly suppresses collagen-, thrombin-, and AA-induced platelet activations by inhibiting phosphorylations of Syk and PLCγ2. In order to further illuminate the underlying mechanism of XML-mediated inhibition of platelet activation, we examined the phosphorylation of MAPKs (ERK, JNK, and p38) and found that XML inhibited collagen-, thrombin-, and AA-induced phosphorylations of ERK1/2, JNK, and p38. Moreover, XML also inhibited collagen-, thrombin-, and AA-induced phosphorylations of Akt and GSK3β, indicating that XML also functions as a repressor of PI3K-Akt-GSK3β signaling to attenuate platelet activation. Therefore, XML acted as inhibitor of both the GPVI-Syk-PLCγ2-MAPK and PI3K-Akt-GSK3β signaling pathways. Furthermore, the additive inhibitory effect of XML and PP2 or LY294002 on collagen-induced platelet aggregation indicates that XML inhibited simultaneously both SFK-initiated signaling and PI3K-based signaling. Our results demonstrated that agonist-induced integrin α II bβ3 “inside-out” signaling was inhibited by XML.

Moreover, fibrinogen binding to activated integrin α II bβ3 triggers “outside-in” signaling events, inducing integrin clustering that leads to cytoskeletal reorganization and post-occupancy events including platelet spreading, platelet aggregation, and clot retraction (Boylan et al., 2008; Vaiyapuri et al., 2013). We employed these well-accepted assays and found that XML robustly suppressed platelet spreading on fibrinogen-coated surfaces and clot retraction, indicating that XML can block integrin α II bβ3-mediated “outside-in” signaling.

We extended the study to the *in vivo* setting and examined the effect of XML on thrombus formation using an acute pulmonary thrombosis mouse model, which involves the formation of thrombi in the lung vasculature. We found that XML-treated mice were protected from lung vascular thrombosis. Although XML showed anti-platelet and anti-thrombotic effect, it did not prolonged mouse tail-bleeding time; neither PT nor APTT was affected by XML, indicating XML has no effect on the coagulation system.

However, our study has limitations. Due to the complex active ingredients of XML and multiple signaling pathways of antiplatelet, we are still far away from fully known the mechanisms of the anti-platelet and anti-thrombotic. Further studies are required to decipher other mechanisms of its function in platelet function and thrombus formation.

In summary, the results of present study demonstrate that XML potently suppresses aggregation and granule secretion induced by several agonists. Additionally, our results show that XML negatively regulates platelet adhesion and spreading on both collagen- and fibrinogen-coated surfaces. Furthermore, we show that XML inhibits platelet activation by inhibiting signaling pathways including the GPVI-Syk-PLCγ2-MAPK and PI3K-Akt-GSK3β pathways, followed by suppression of integrin α II bβ3-mediated signaling. Finally, we report that XML protects mice from thrombosis formation in the acute lung thromboembolism model. Collectively, our results suggest that XML may be a potential antiplatelet and anti-thrombotic agent.

## Data Availability

All datasets generated for this study are included in the manuscript and the **Supplementary Files**.

## Ethics Statement

The study protocol was approved by the Ethics Committee of Kunming Medical University in accordance with the Helsinki Declaration for the Use of Human Subjects. All volunteers signed informed consent.

The protocol for the in vivo study with rats was reviewed and approved by the Ethics Committee for experimental animals, Kunming Medical University.

## Author Contributions

LW and ZM designed the experiments. HW, YY, WW, and LL carried out the experiments. RL and LD analyzed the experimental results. LY, LHY, and YG wrote the manuscript.

## Funding

This work was supported by the National Natural Science Foundation of China (No. 81160025, 81560075, 81860074 and 81860073).

## Conflict of Interest Statement

The authors declare that the research was conducted in the absence of any commercial or financial relationships that could be construed as a potential conflict of interest.
