# Supplementary material for: Xinmailong Modulates Platelet Function and Inhibits Thrombus Formation via the Platelet αIIbβ3-Mediated Signaling Pathway
Source: Front Pharmacol. 2019 Aug 23;10:923. doi: 10.3389/fphar.2019.00923 (PMC6716460; doi:10.3389/fphar.2019.00923)
Supplement: Supplementary file 1 [file DataSheet_1.zip › uncropped image of western blots/2/illustration.docx]

1 2 3 4 5 6 7 8 9 10 11 12

1: Collagen 1μg/ml (-), XML 10mg/ml (-)

2: Collagen 1μg/ml (+), XML 10mg/ml (-)

3: Collagen 1μg/ml (+), XML 10mg/ml (+)

4: Thrombin 0.08U/L (-), XML 10mg/ml (-)

5: Thrombin 0.08U/L (+), XML 10mg/ml (-)

6: Thrombin 0.08U/L (+), XML 10mg/ml (+)

7: AA 100μM (-), XML 10mg/ml (-)

8: AA 100μM (+), XML 10mg/ml (-)

9: AA 100μM (+), XML 10mg/ml (+)

10: AA 100μM (-), XML 10mg/ml (-)

11: AA 100μM (+), XML 10mg/ml (-)

12: AA 100μM (+), XML 10mg/ml (+)
