# Supplementary figures and images for: Xinmailong Modulates Platelet Function and Inhibits Thrombus Formation via the Platelet αIIbβ3-Mediated Signaling Pathway
Source: Front Pharmacol. 2019 Aug 23;10:923. doi: 10.3389/fphar.2019.00923 (PMC6716460; doi:10.3389/fphar.2019.00923)

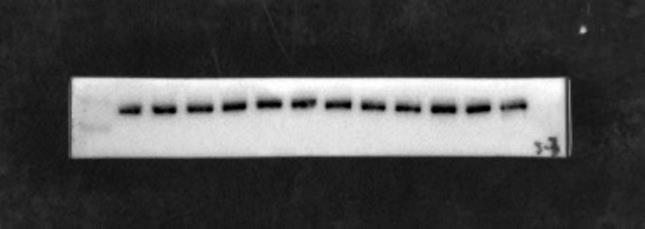

Supplement: Supplementary file 1 [file DataSheet_1.zip › uncropped image of western blots/1/GAPDH.jpg]

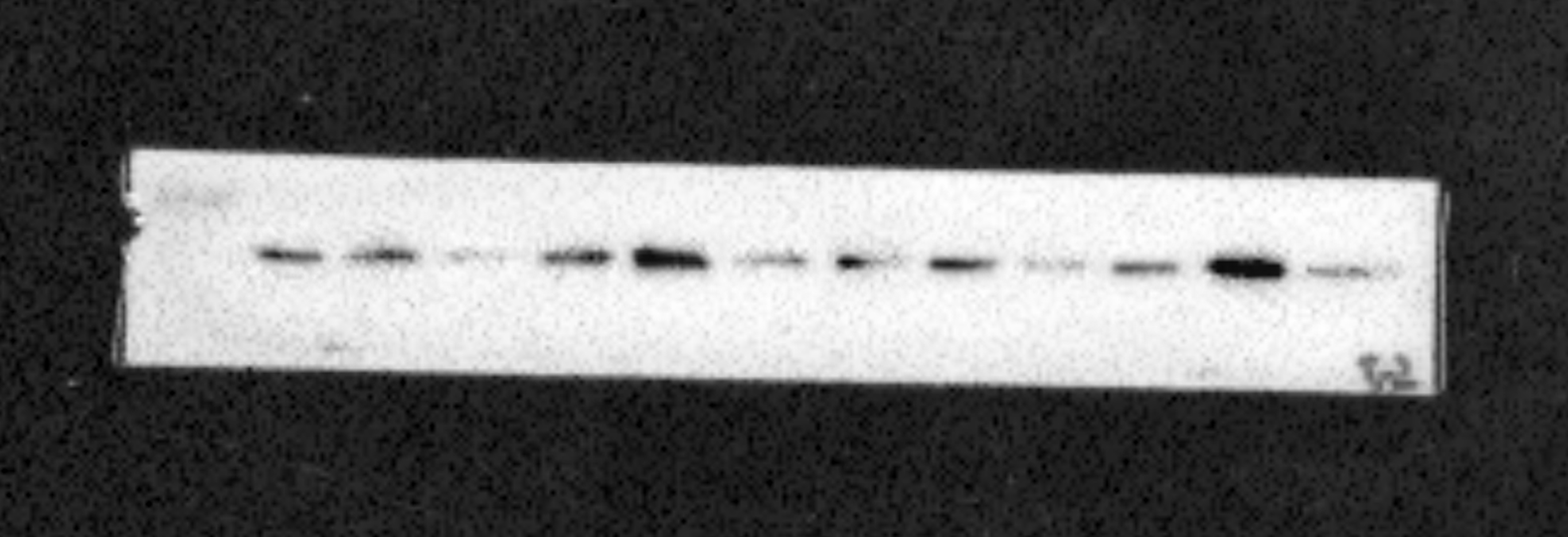

Supplement: Supplementary file 1 [file DataSheet_1.zip › uncropped image of western blots/1/P-Akt.tif]

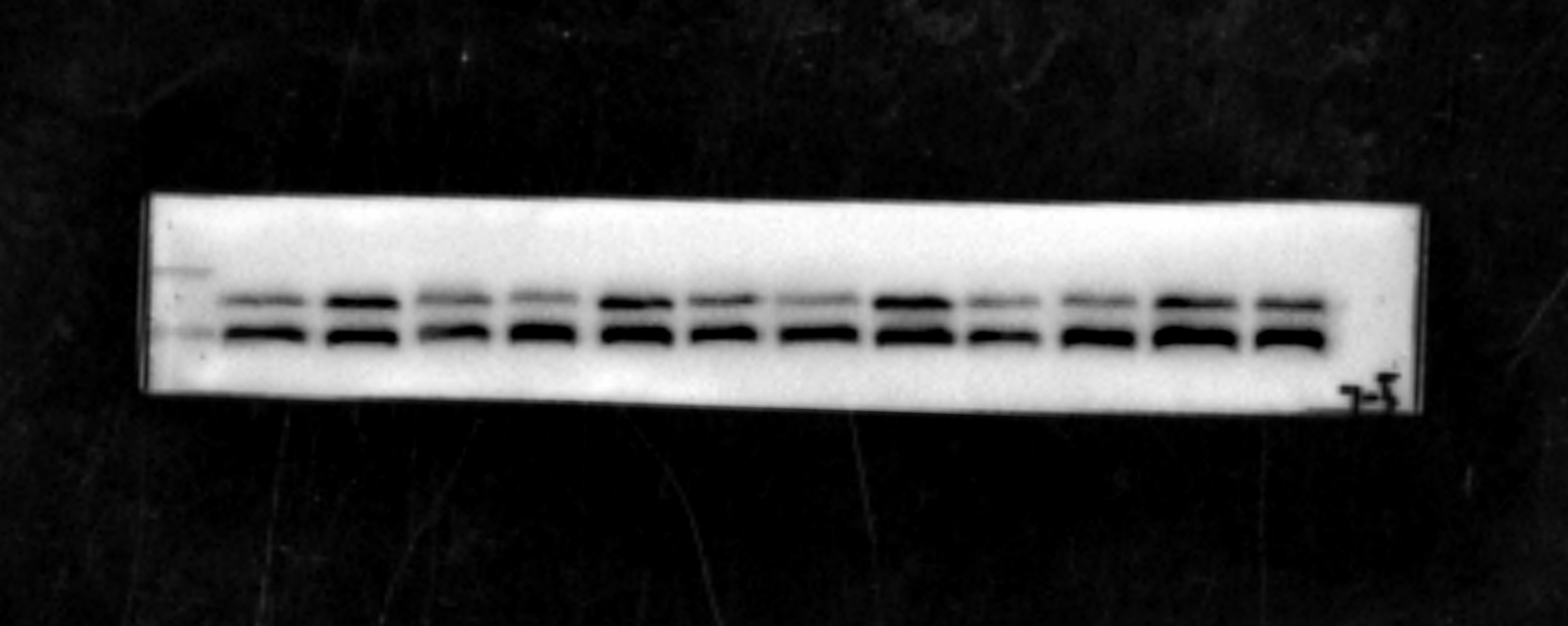

Supplement: Supplementary file 1 [file DataSheet_1.zip › uncropped image of western blots/1/P-Erk .tif]

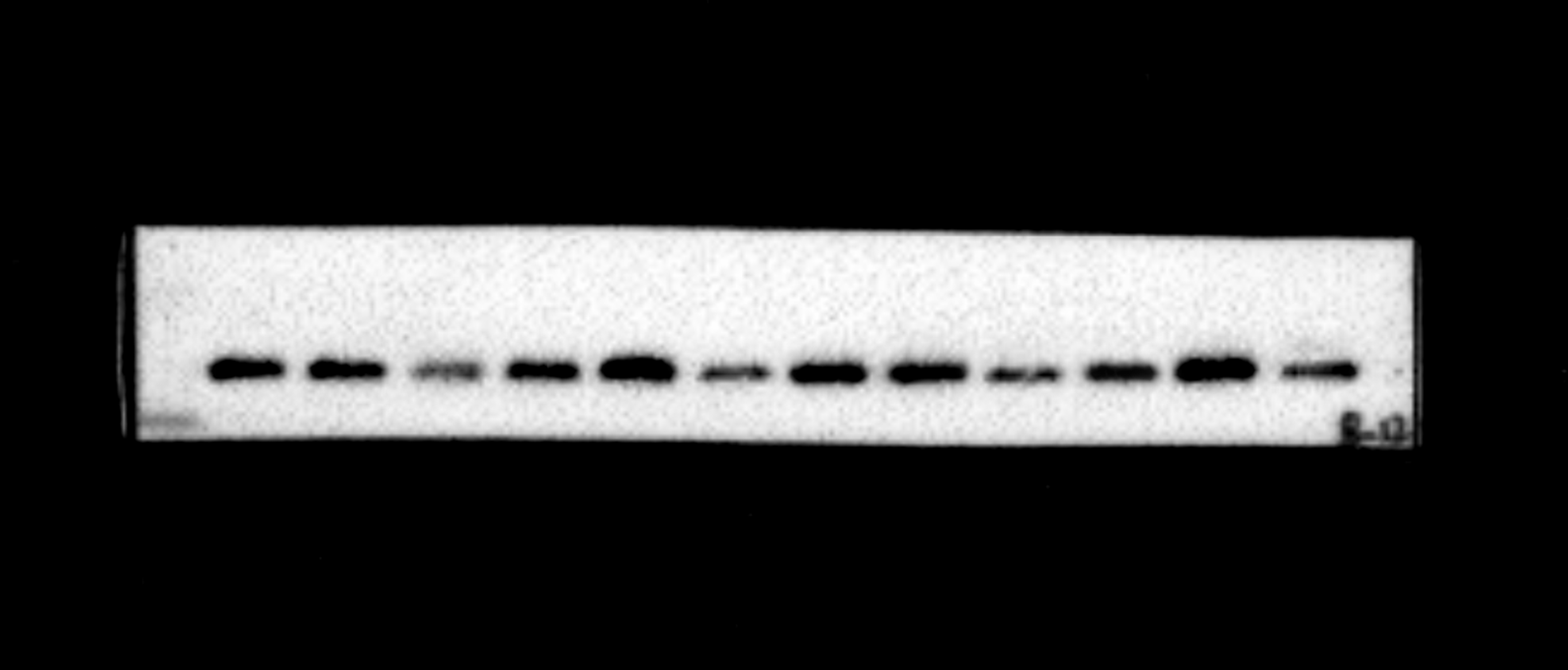

Supplement: Supplementary file 1 [file DataSheet_1.zip › uncropped image of western blots/1/P-GSK3β.tif]

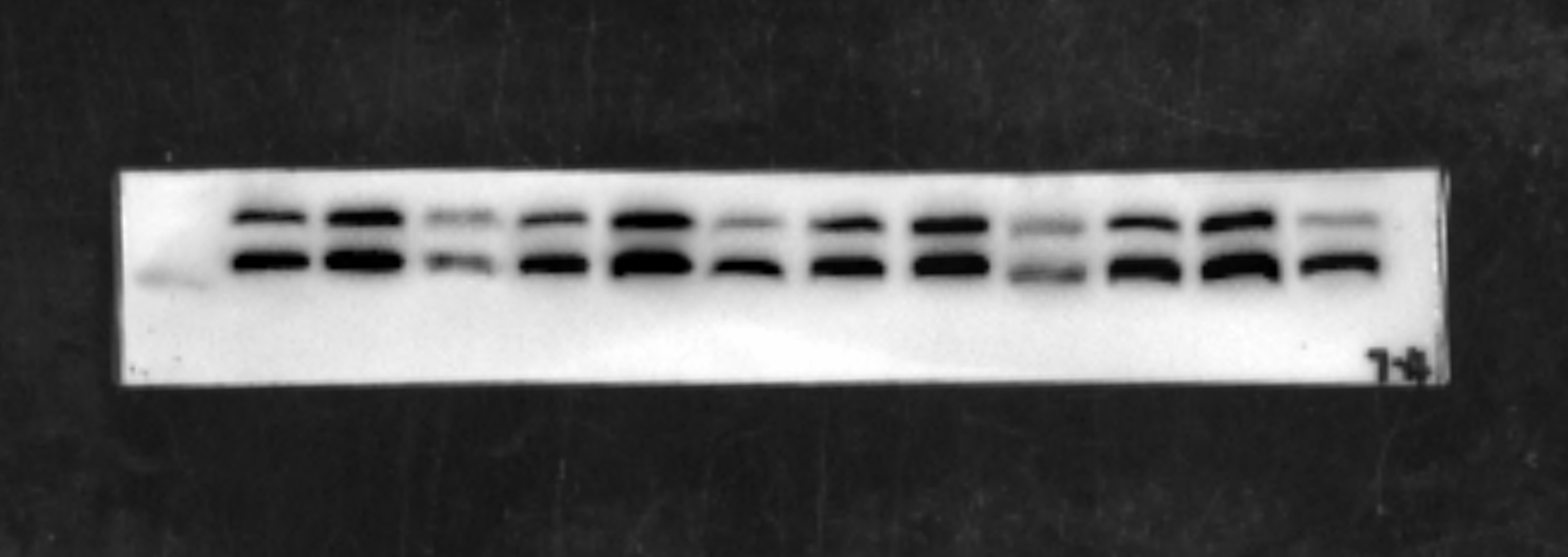

Supplement: Supplementary file 1 [file DataSheet_1.zip › uncropped image of western blots/1/P-JNK.tif]

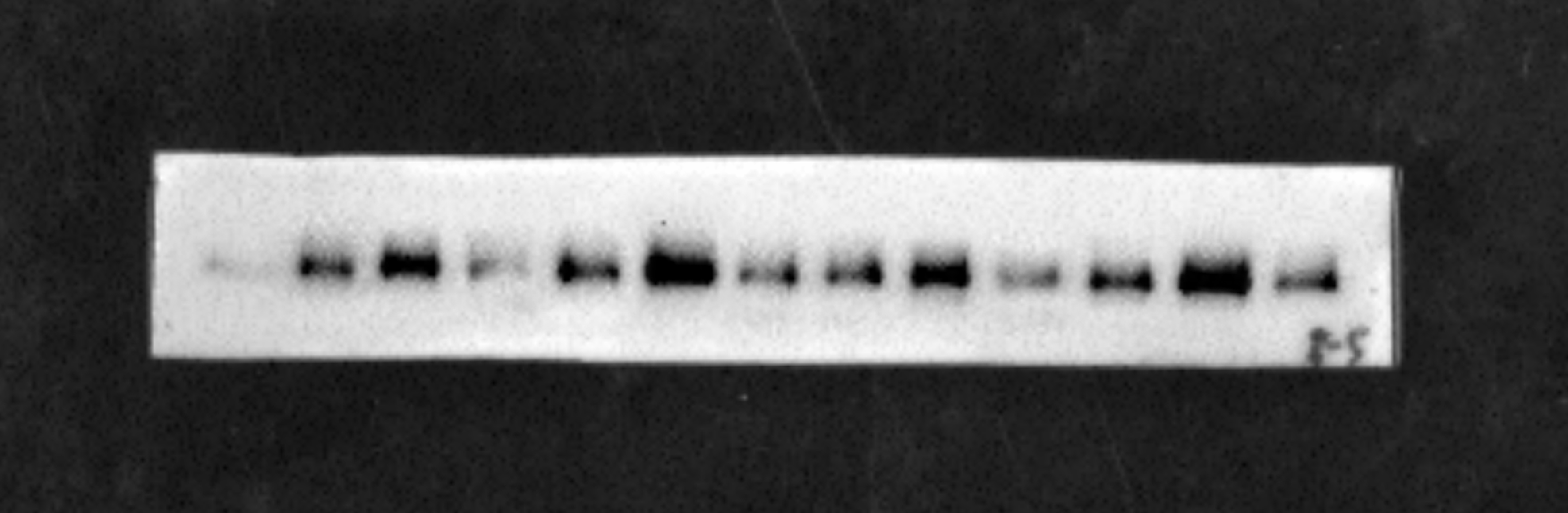

Supplement: Supplementary file 1 [file DataSheet_1.zip › uncropped image of western blots/1/P-p38.tif]

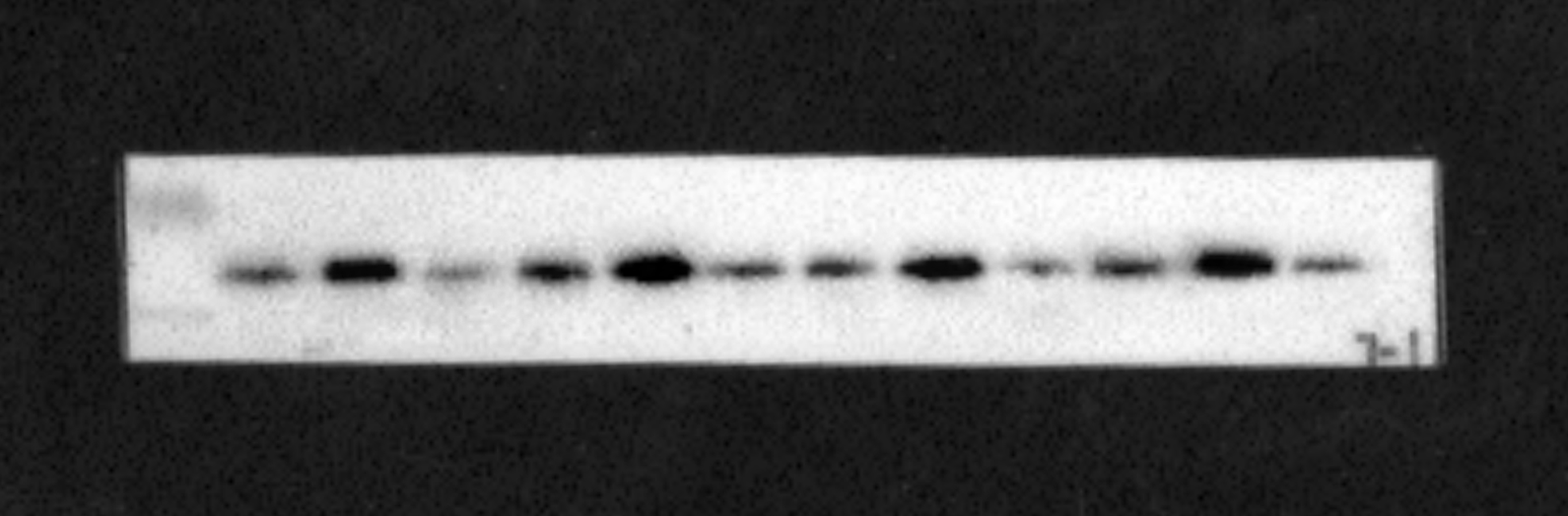

Supplement: Supplementary file 1 [file DataSheet_1.zip › uncropped image of western blots/1/P-PLCγ2.tif]

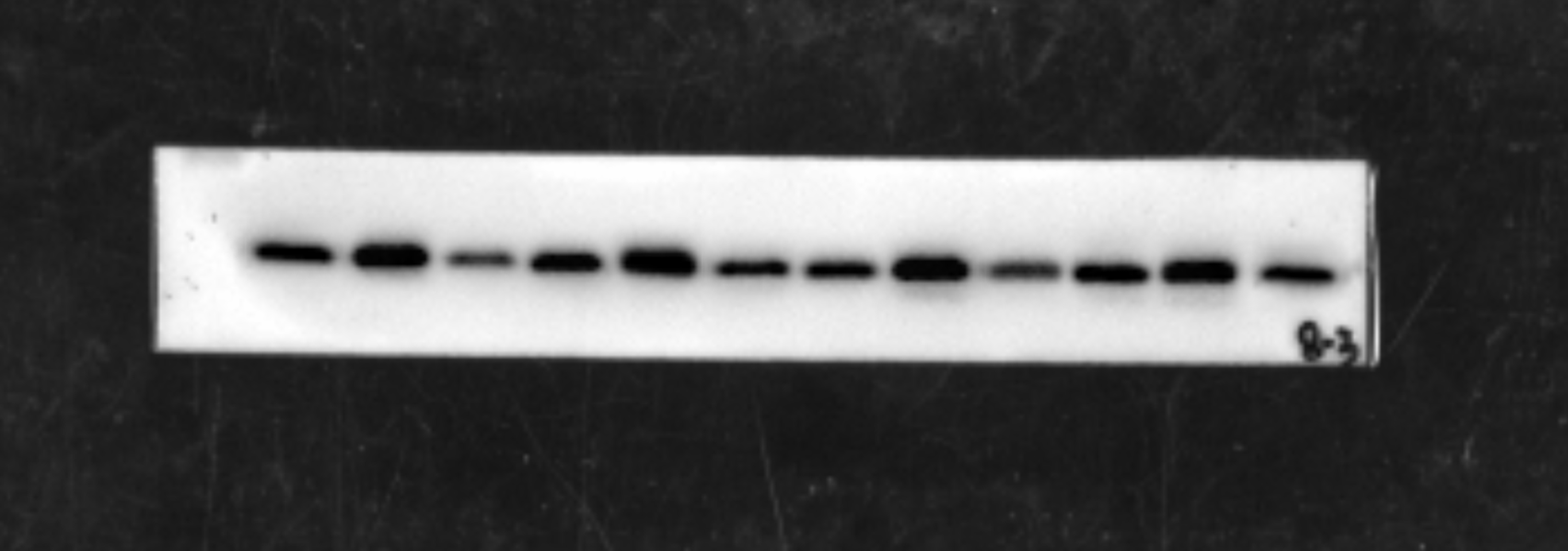

Supplement: Supplementary file 1 [file DataSheet_1.zip › uncropped image of western blots/1/P-Syk.tif]

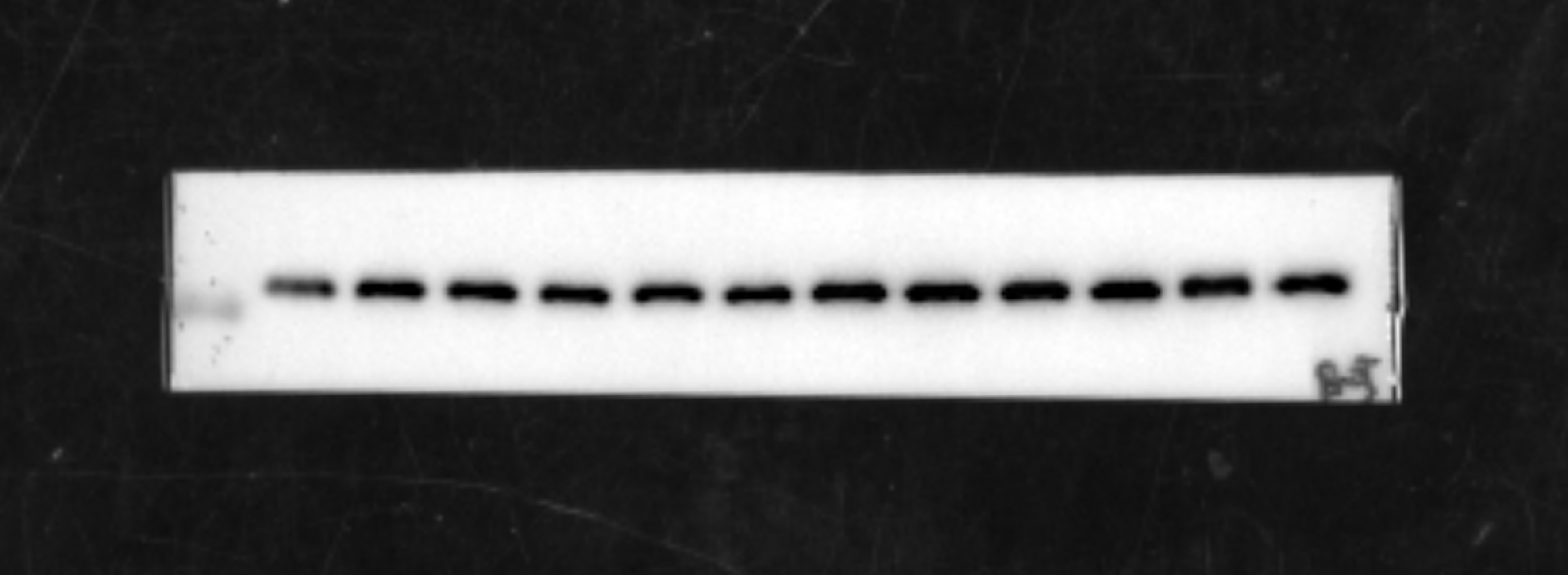

Supplement: Supplementary file 1 [file DataSheet_1.zip › uncropped image of western blots/1/T-Akt.tif]

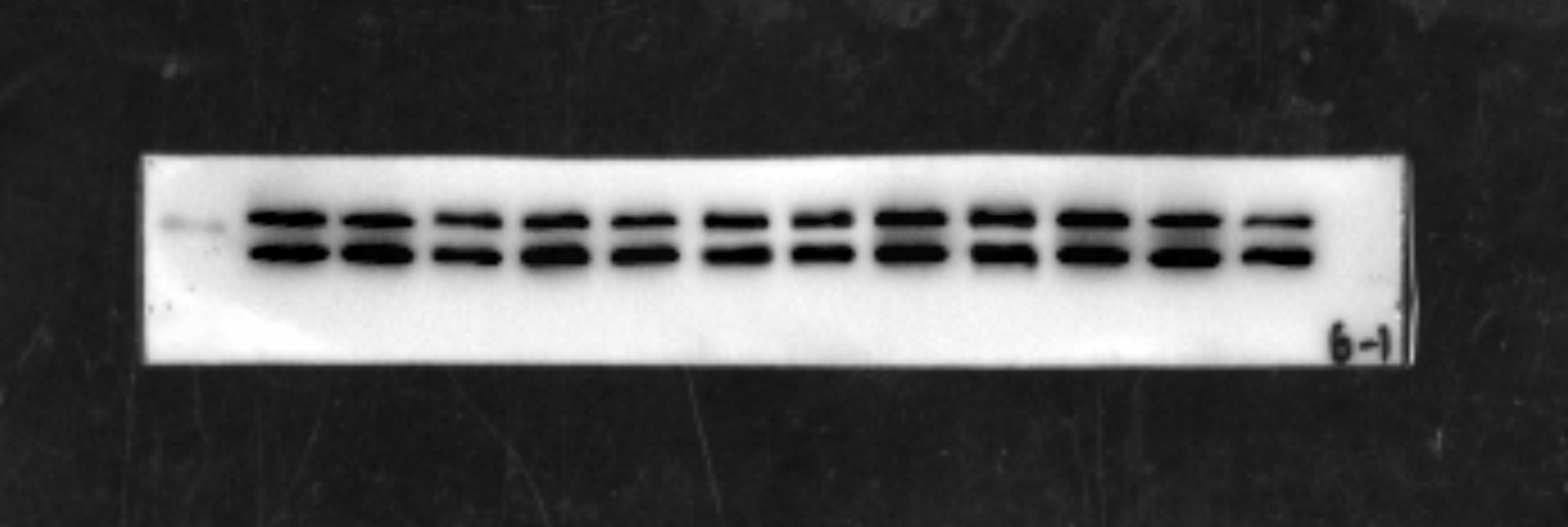

Supplement: Supplementary file 1 [file DataSheet_1.zip › uncropped image of western blots/1/T-Erk.tif]

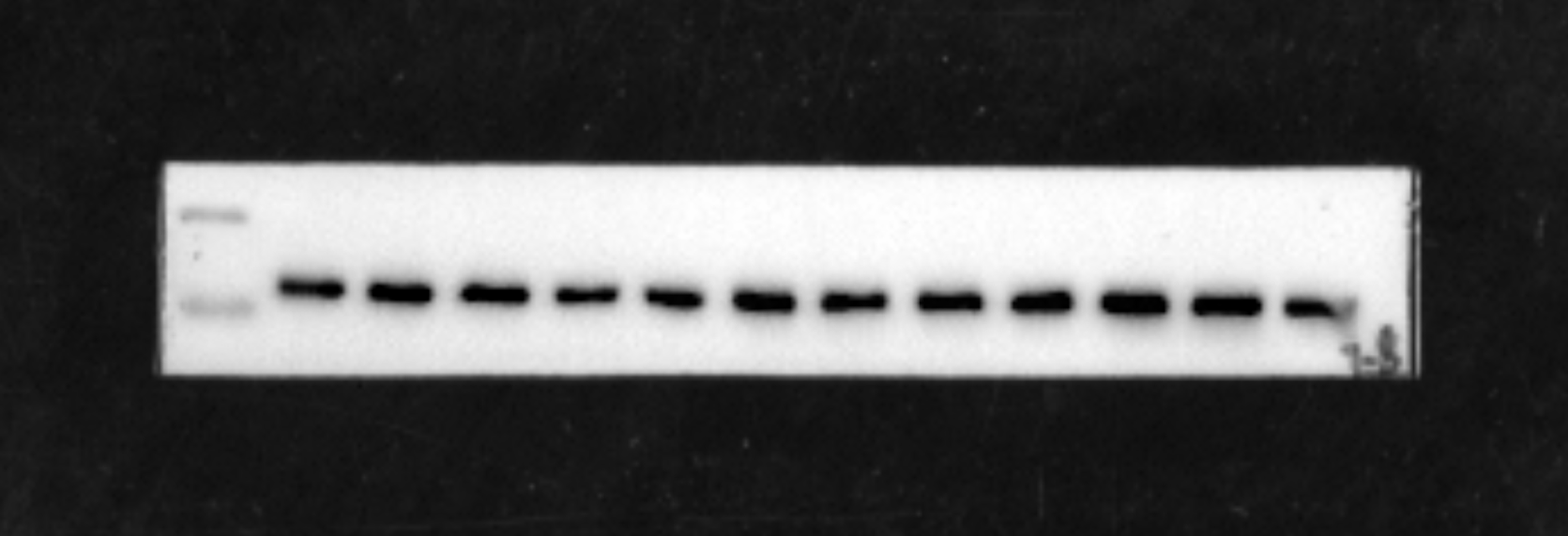

Supplement: Supplementary file 1 [file DataSheet_1.zip › uncropped image of western blots/1/T-GSK3β.tif]

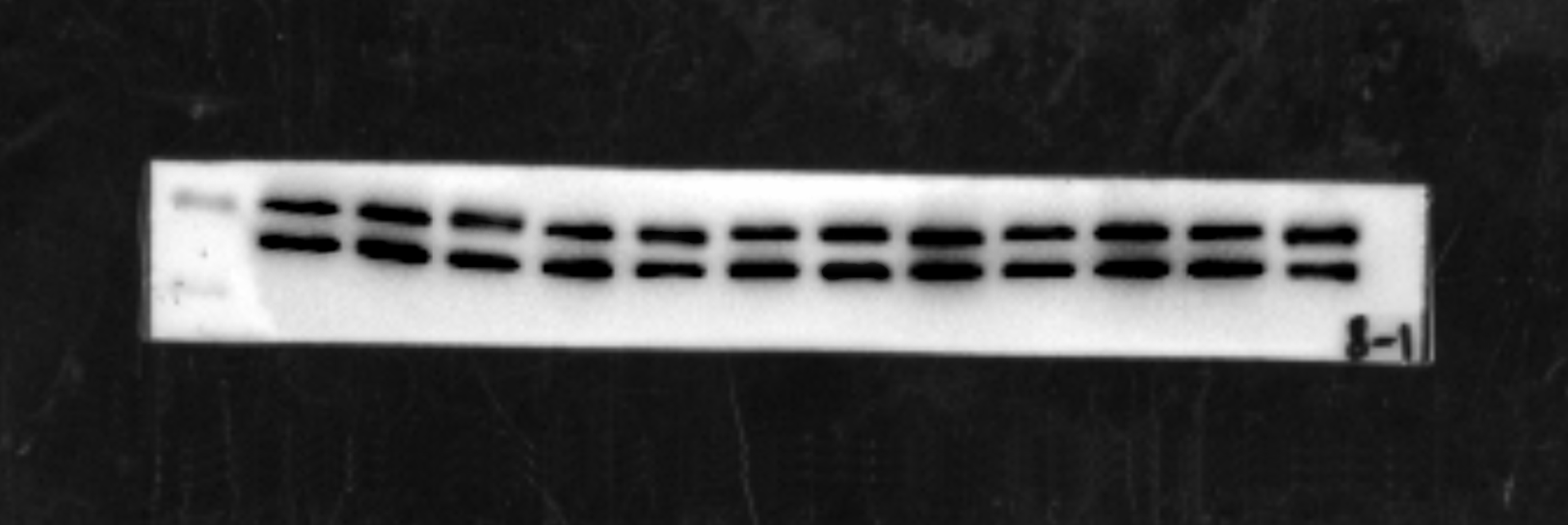

Supplement: Supplementary file 1 [file DataSheet_1.zip › uncropped image of western blots/1/T-JNK.tif]

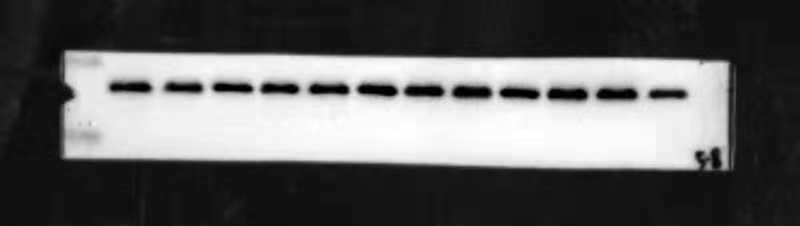

Supplement: Supplementary file 1 [file DataSheet_1.zip › uncropped image of western blots/1/T-p38.jpg]

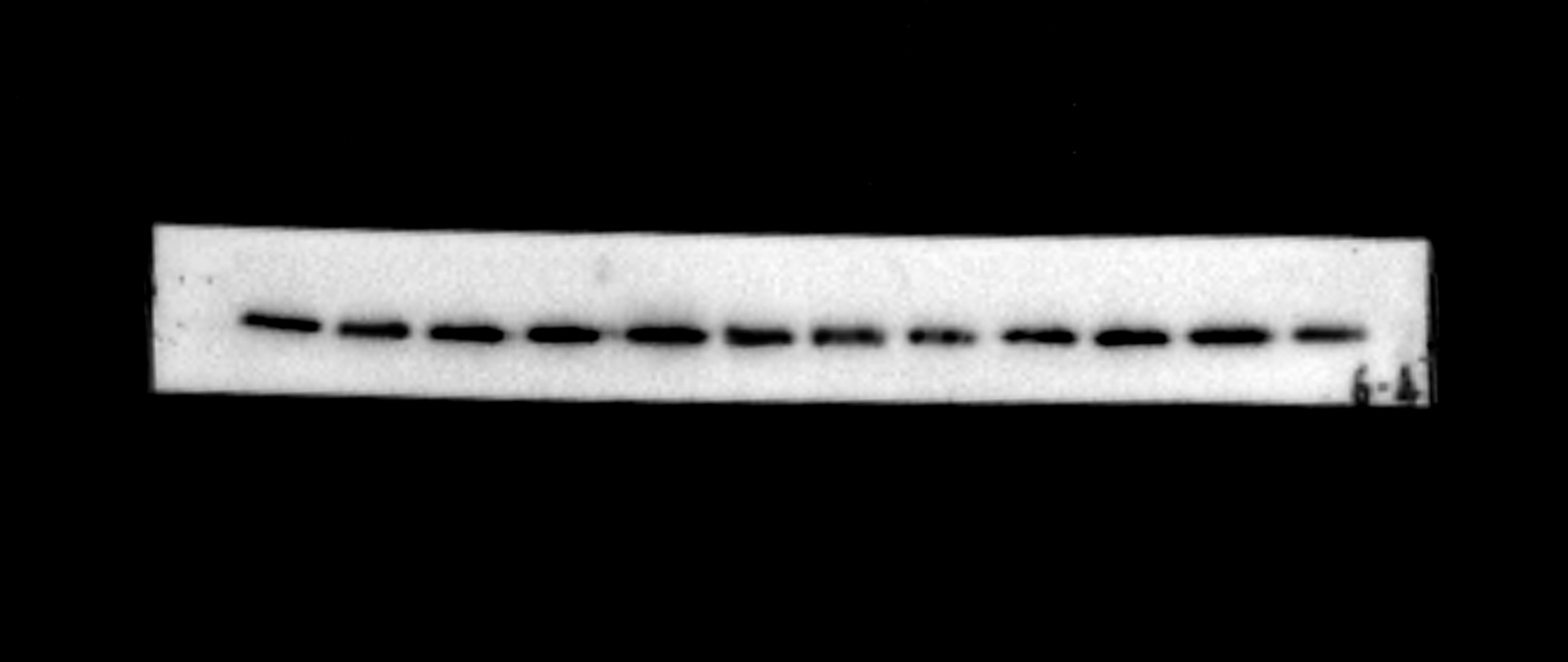

Supplement: Supplementary file 1 [file DataSheet_1.zip › uncropped image of western blots/1/T-PLCγ2.tif]

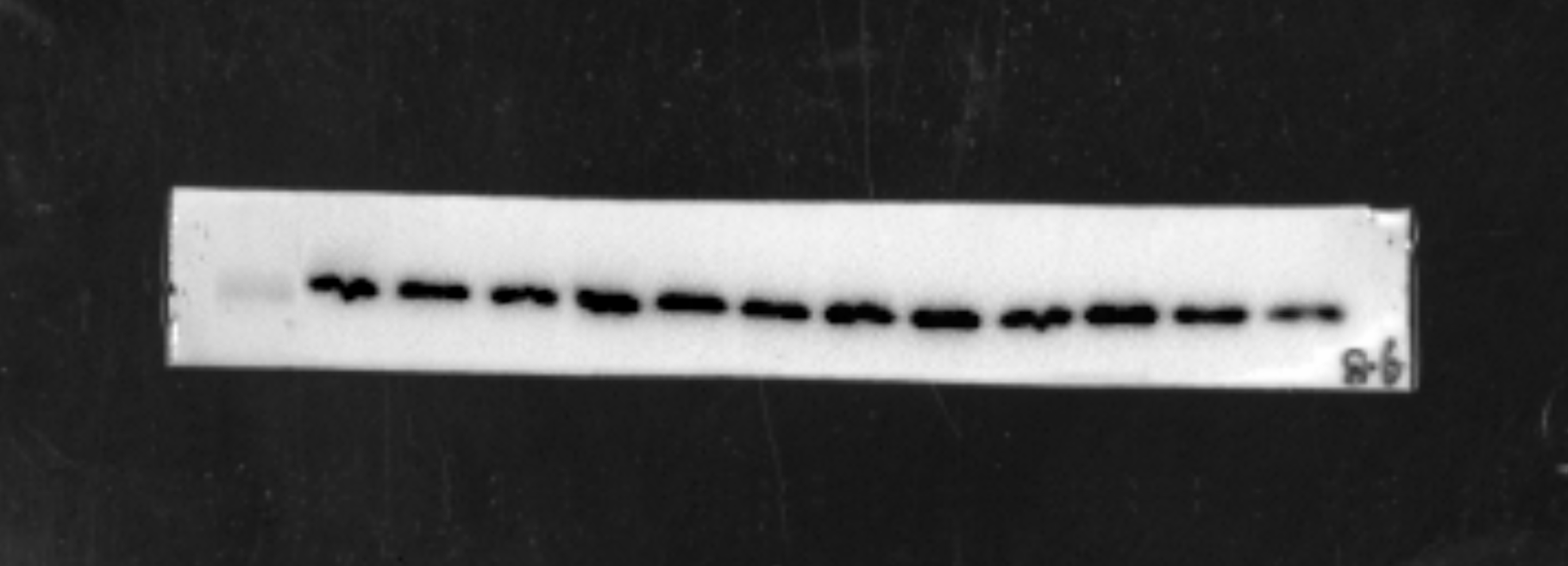

Supplement: Supplementary file 1 [file DataSheet_1.zip › uncropped image of western blots/1/T-Syk.tif]

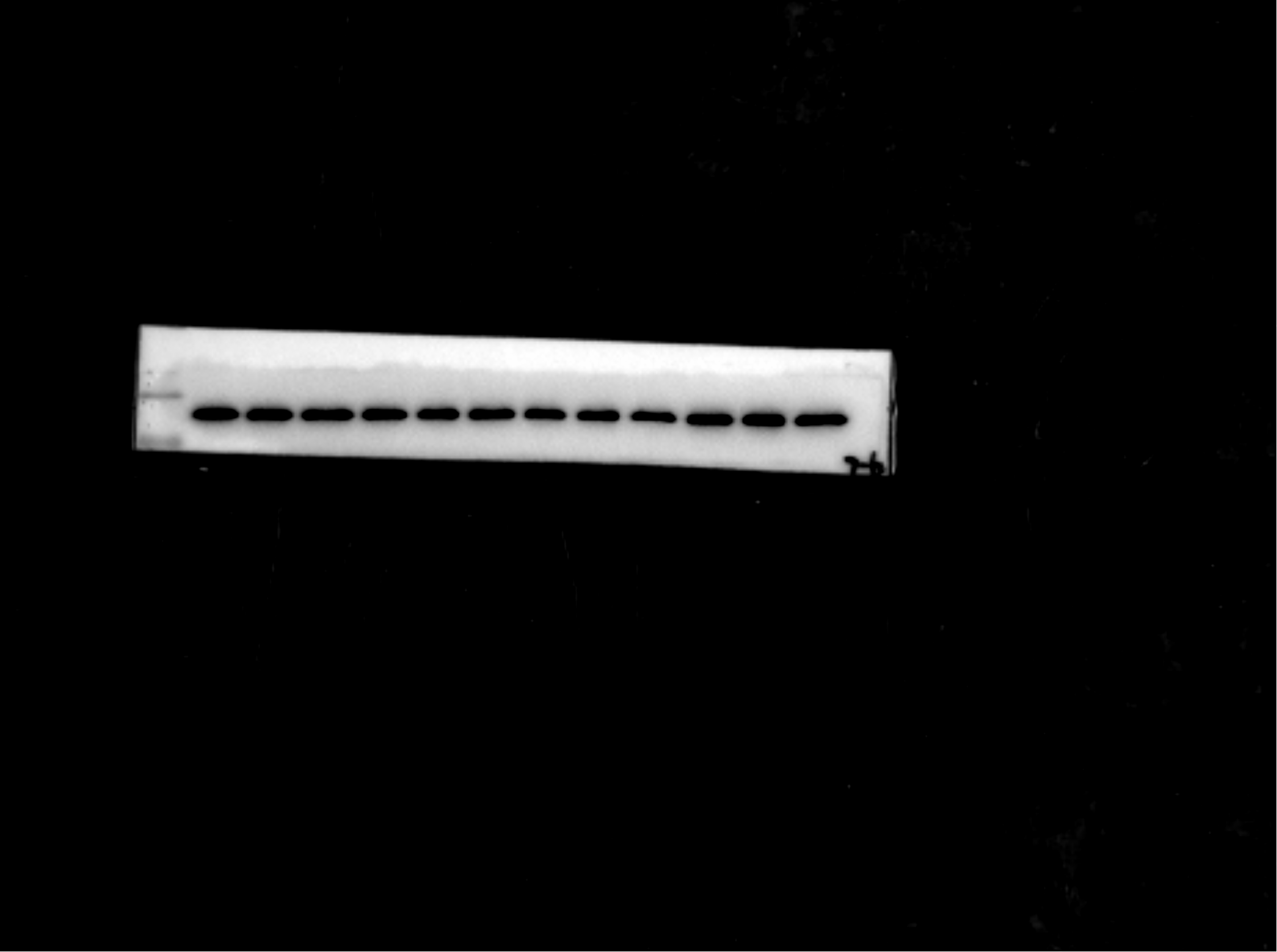

Supplement: Supplementary file 1 [file DataSheet_1.zip › uncropped image of western blots/2/GAPDH .tif]

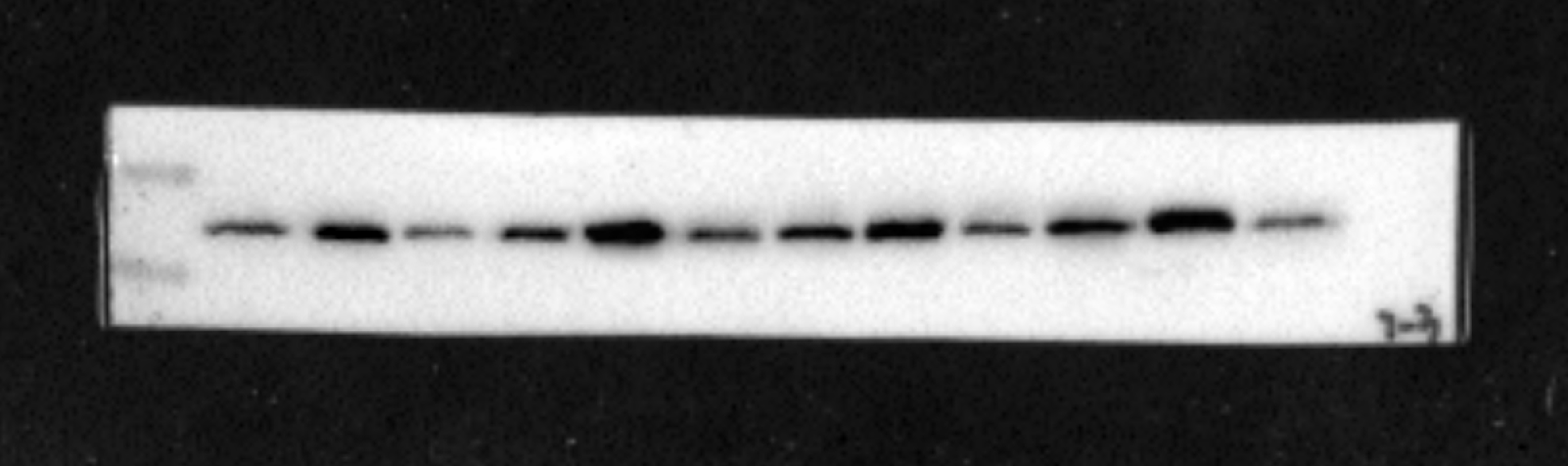

Supplement: Supplementary file 1 [file DataSheet_1.zip › uncropped image of western blots/2/P-Akt.tif]

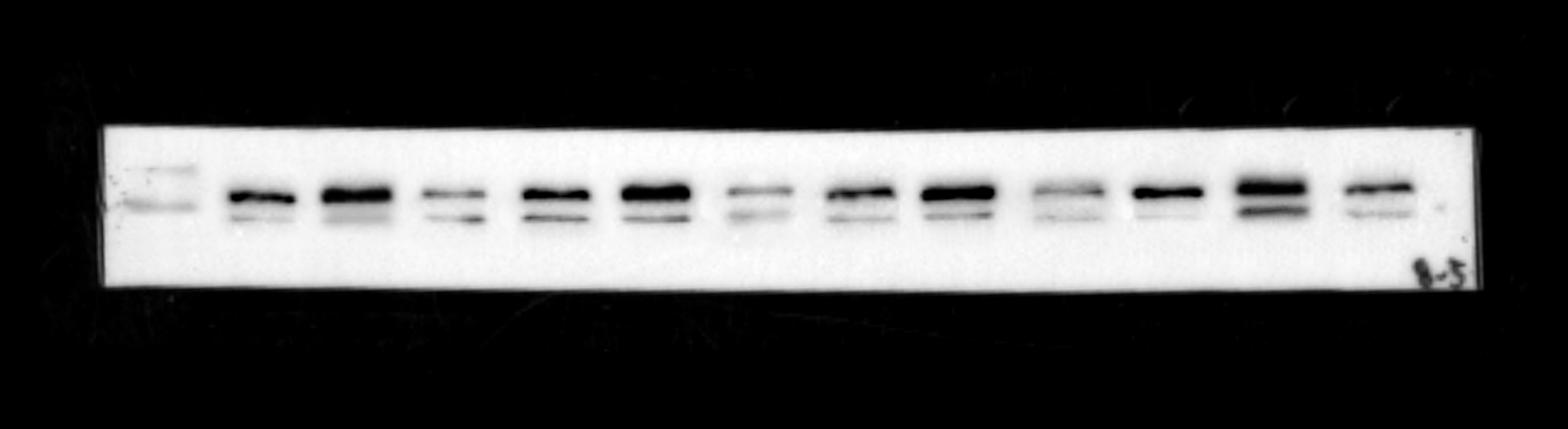

Supplement: Supplementary file 1 [file DataSheet_1.zip › uncropped image of western blots/2/P-ERK.tif]

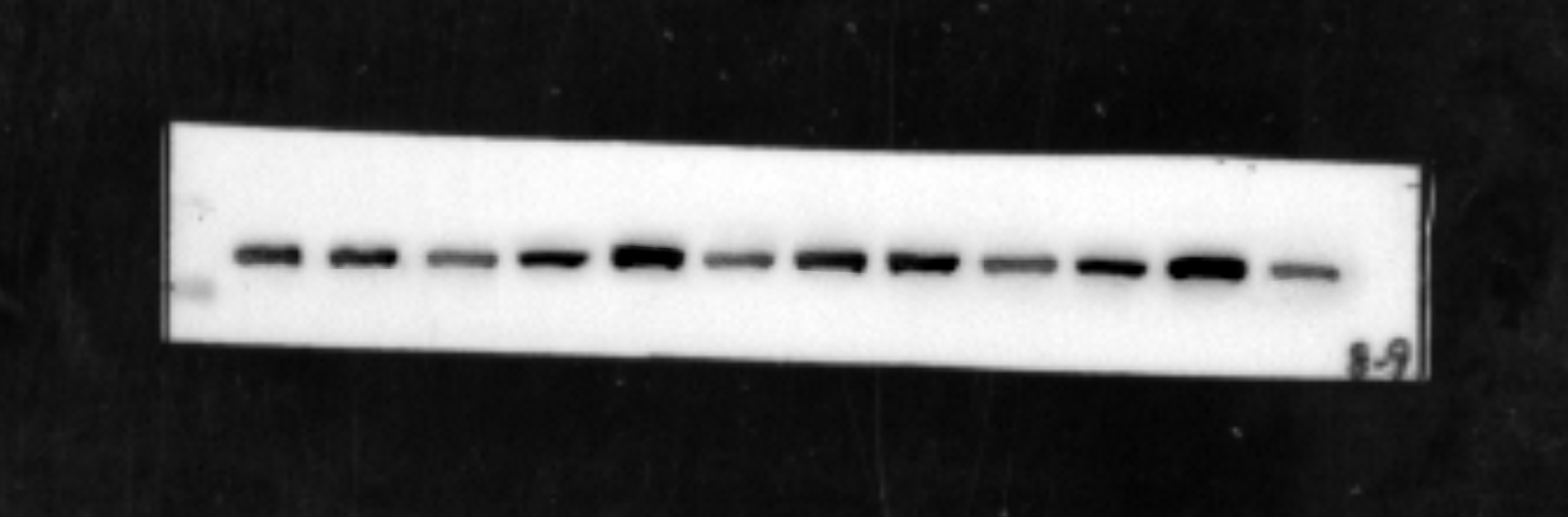

Supplement: Supplementary file 1 [file DataSheet_1.zip › uncropped image of western blots/2/P-GSK3β.tif]

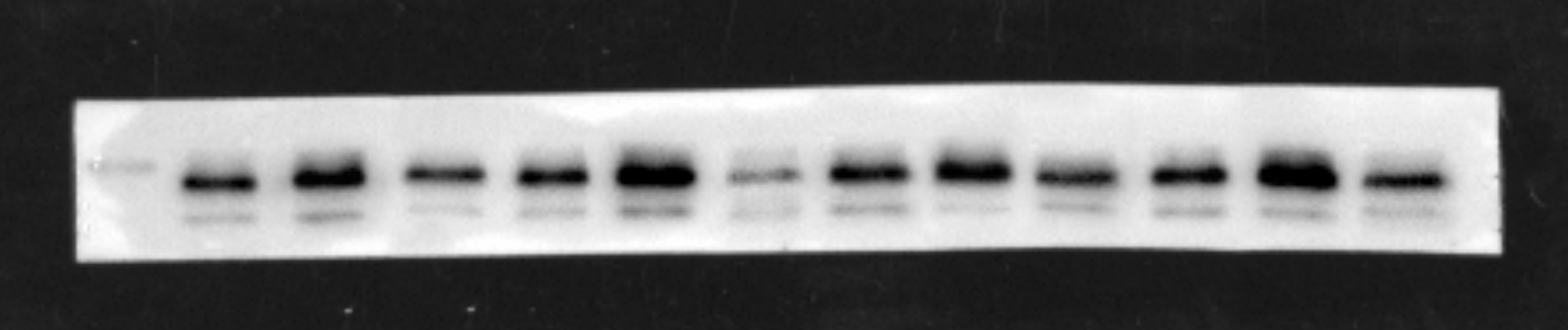

Supplement: Supplementary file 1 [file DataSheet_1.zip › uncropped image of western blots/2/P-JNK.tif]

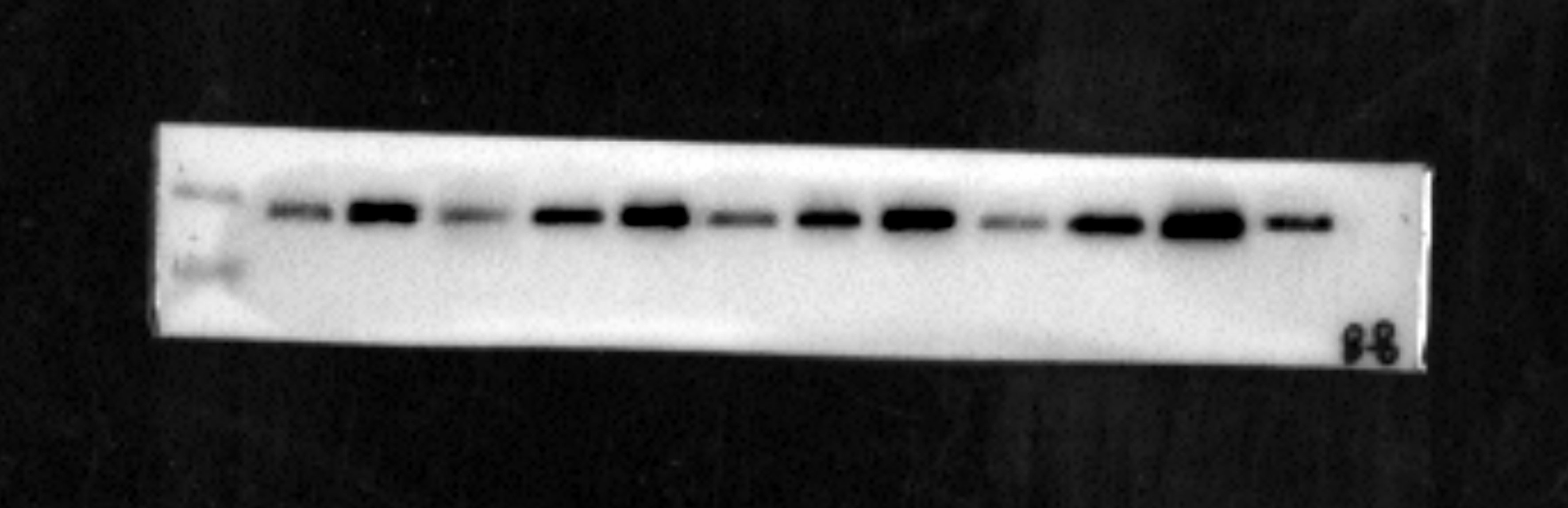

Supplement: Supplementary file 1 [file DataSheet_1.zip › uncropped image of western blots/2/p-p38.tif]

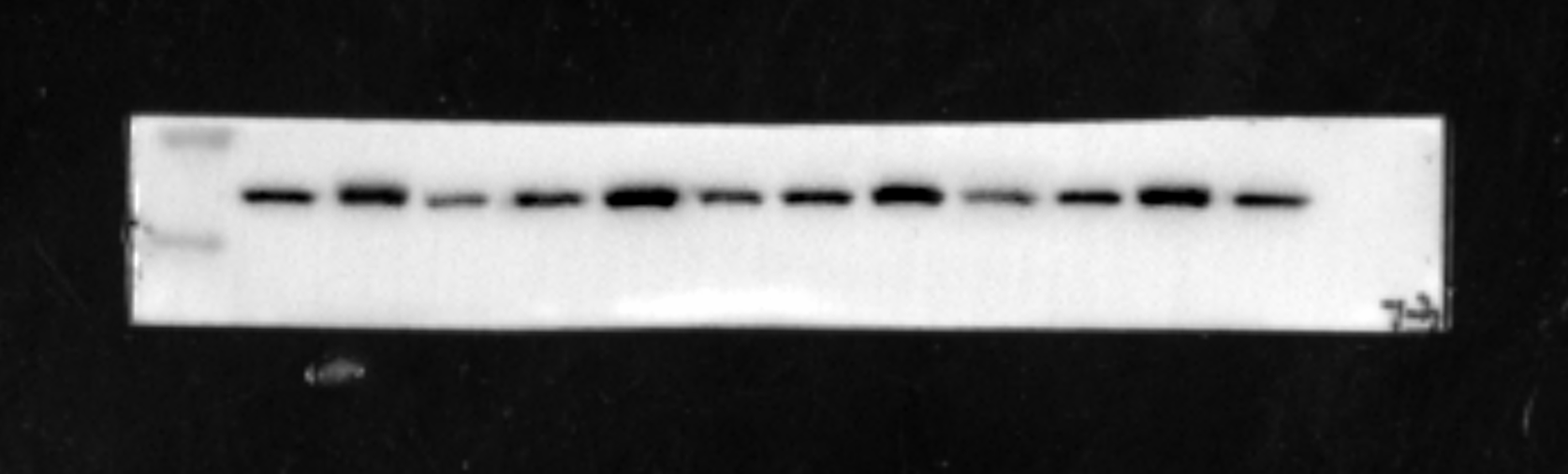

Supplement: Supplementary file 1 [file DataSheet_1.zip › uncropped image of western blots/2/P-PLCγ2.tif]

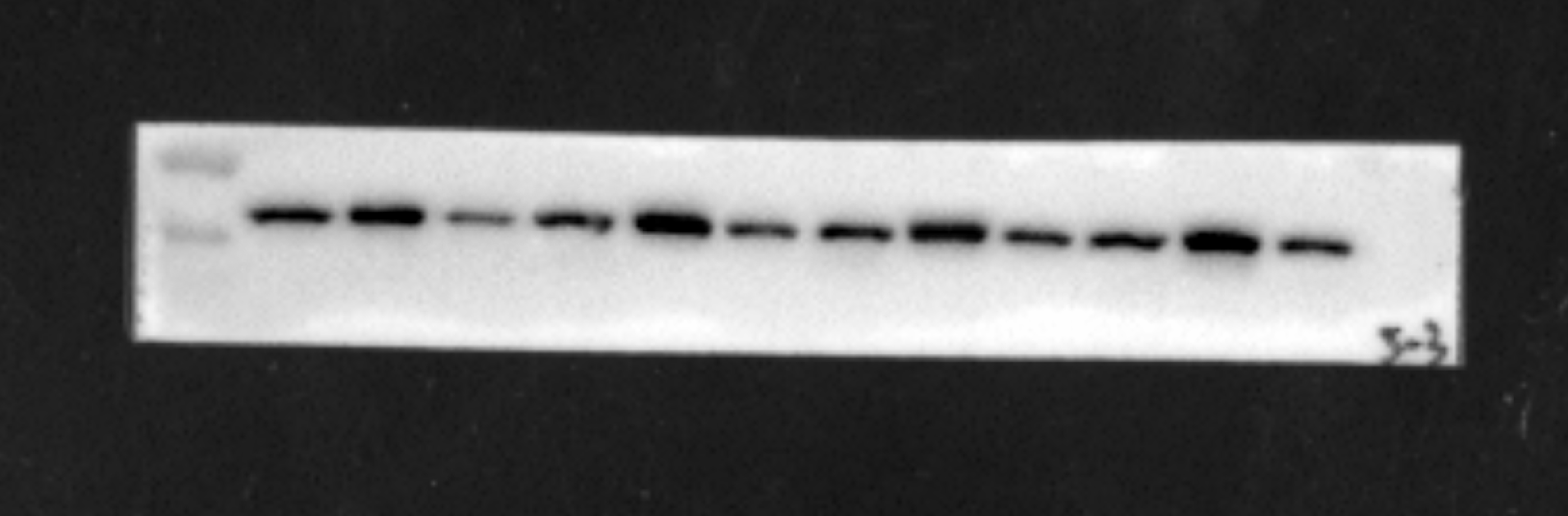

Supplement: Supplementary file 1 [file DataSheet_1.zip › uncropped image of western blots/2/P-Syk.tif]

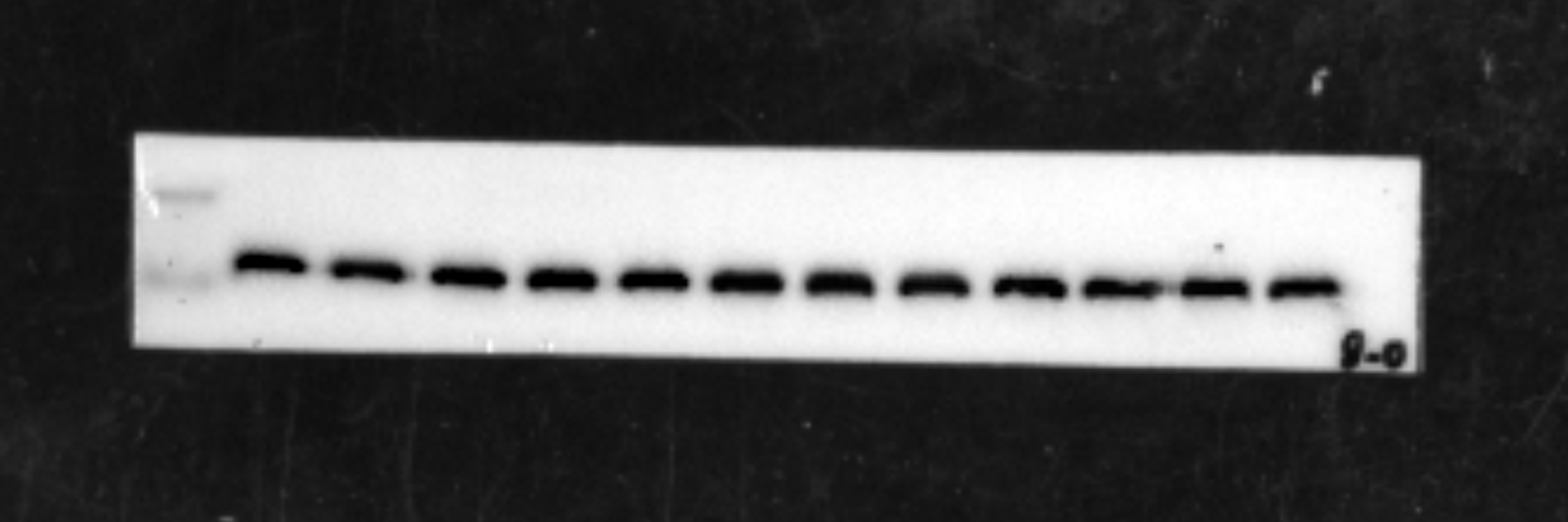

Supplement: Supplementary file 1 [file DataSheet_1.zip › uncropped image of western blots/2/T-Akt.tif]

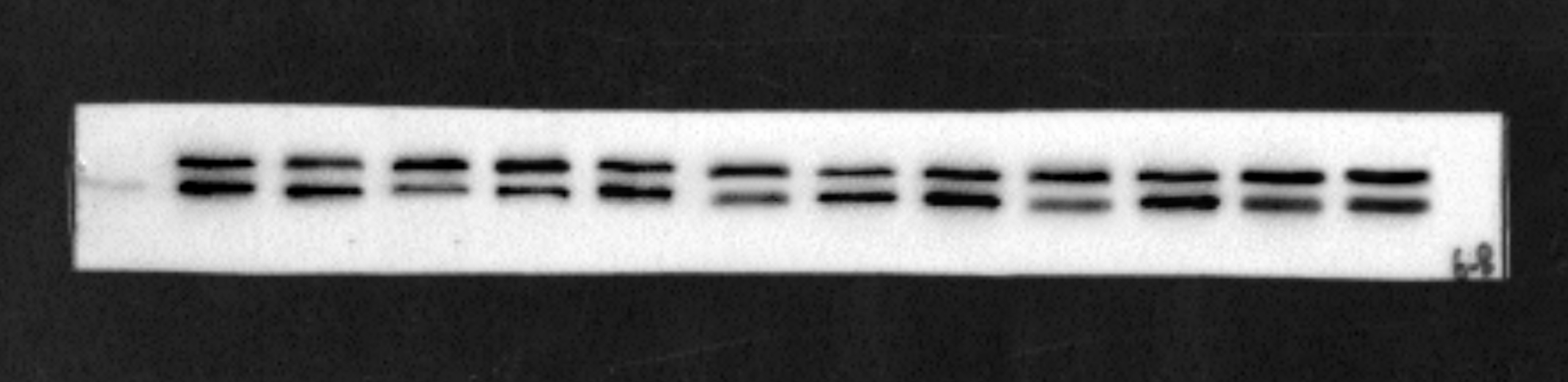

Supplement: Supplementary file 1 [file DataSheet_1.zip › uncropped image of western blots/2/T-ERK.tif]

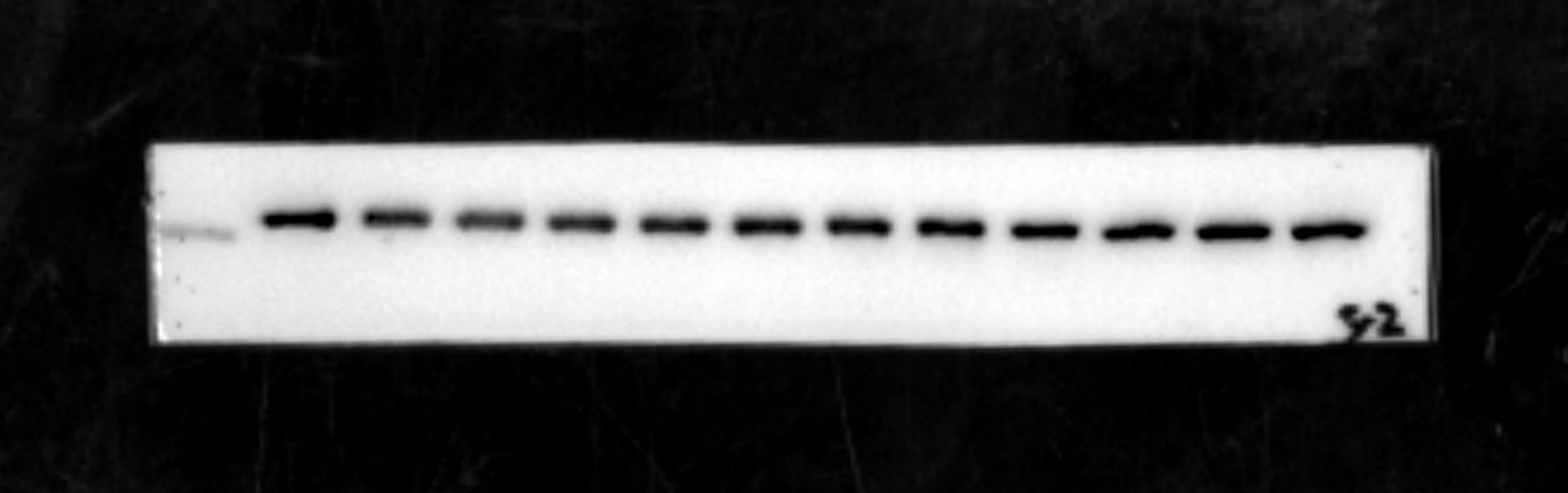

Supplement: Supplementary file 1 [file DataSheet_1.zip › uncropped image of western blots/2/T-GSK3β.tif]

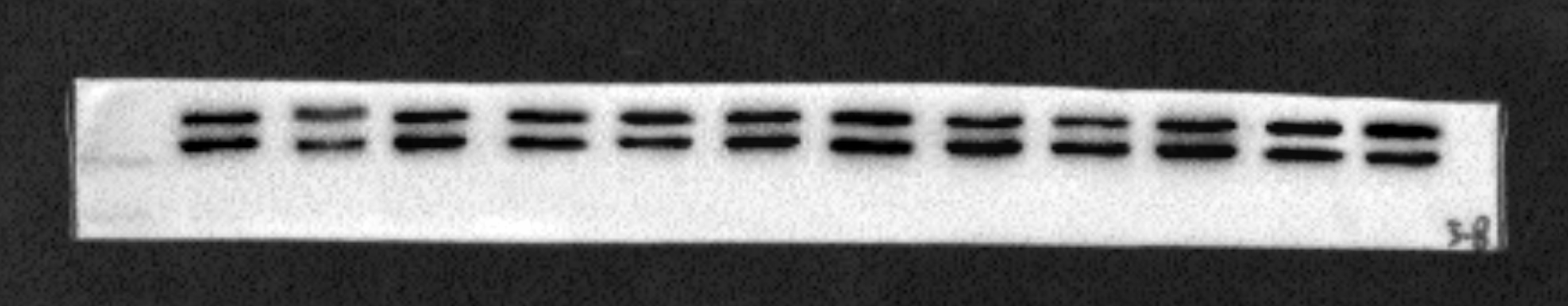

Supplement: Supplementary file 1 [file DataSheet_1.zip › uncropped image of western blots/2/T-JNK.tif]

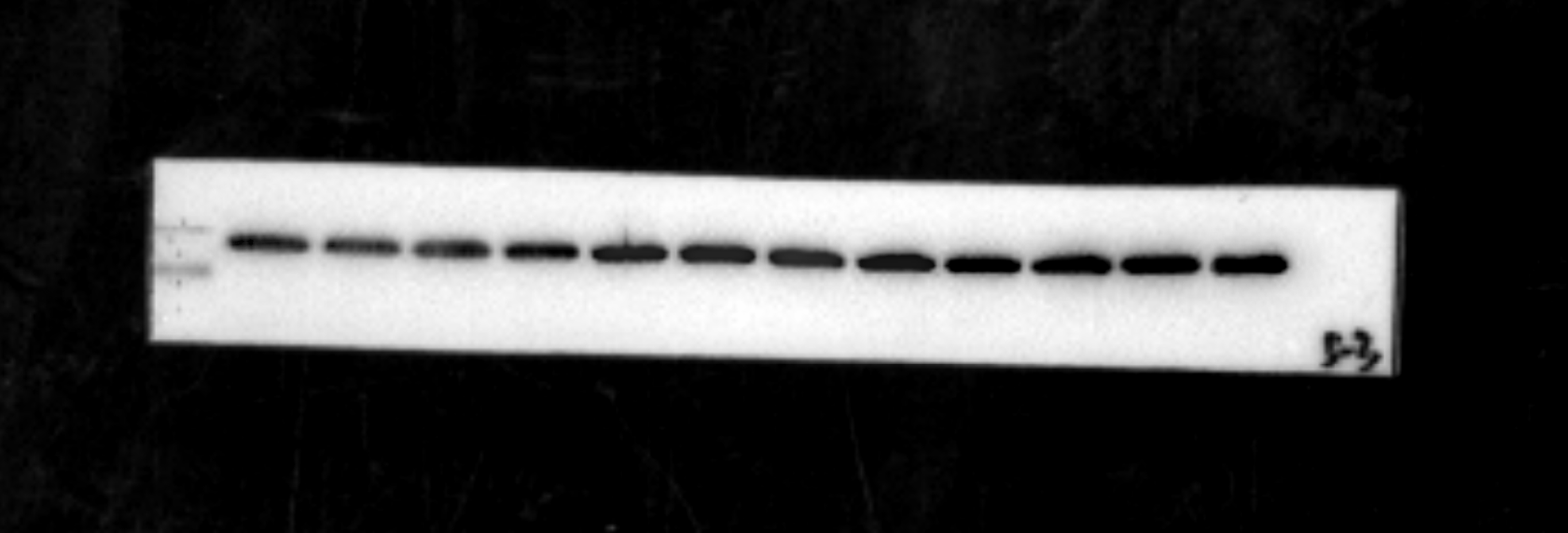

Supplement: Supplementary file 1 [file DataSheet_1.zip › uncropped image of western blots/2/T-p38.tif]

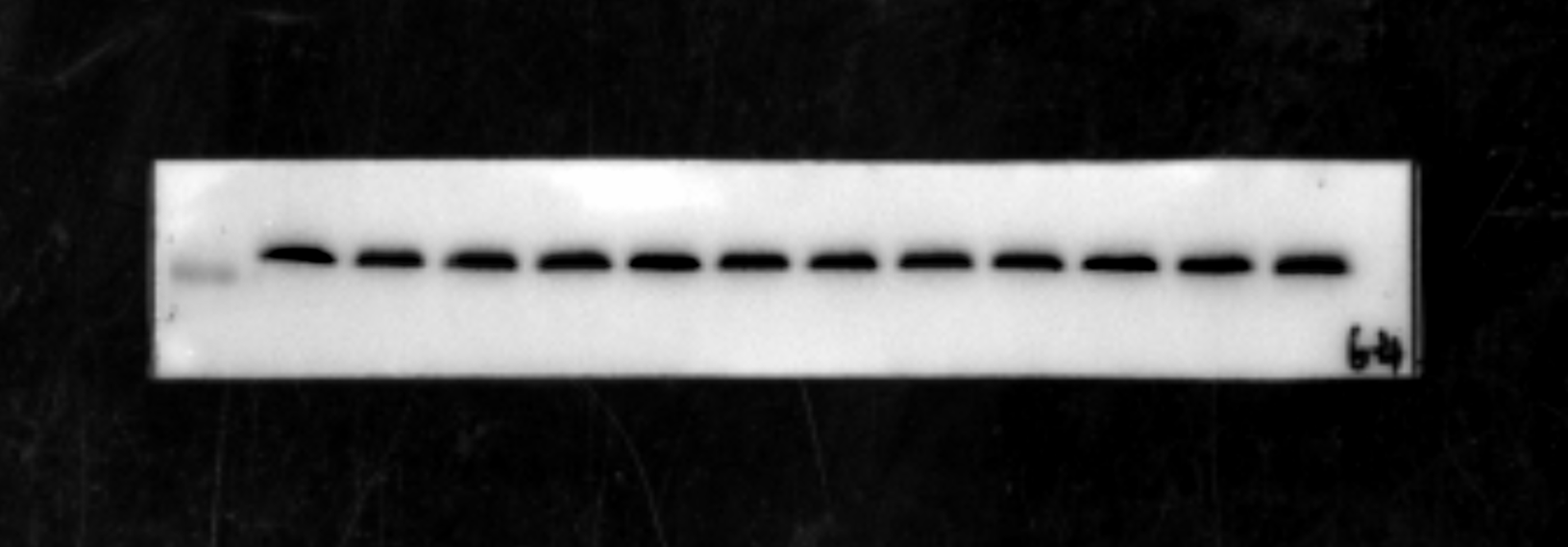

Supplement: Supplementary file 1 [file DataSheet_1.zip › uncropped image of western blots/2/T-PLCγ2.tif]

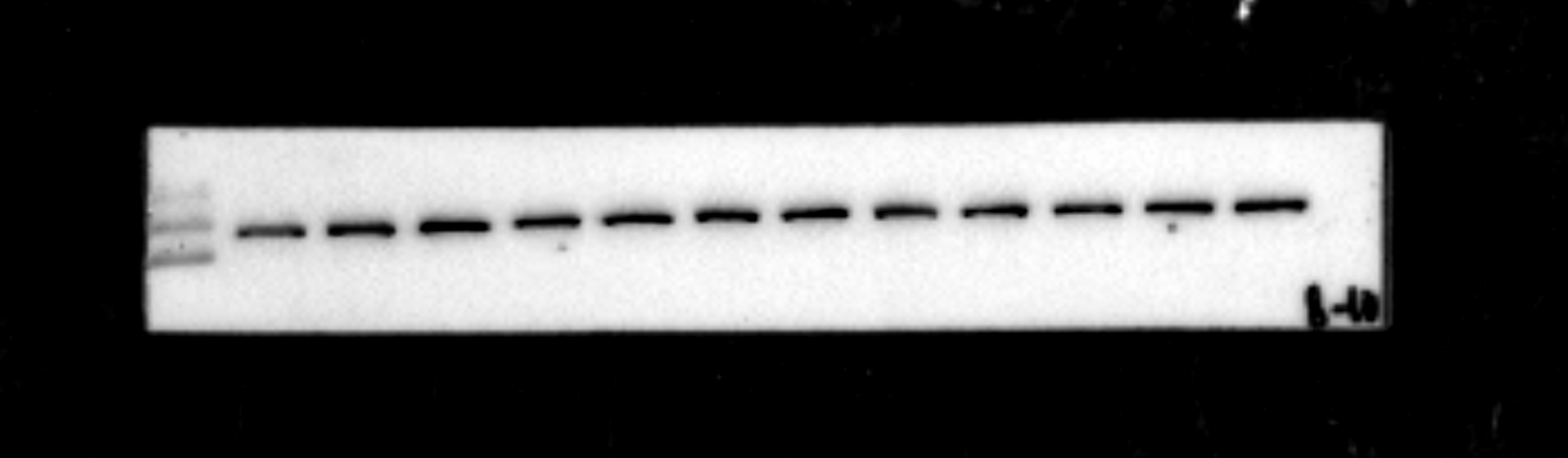

Supplement: Supplementary file 1 [file DataSheet_1.zip › uncropped image of western blots/2/T-Syk.tif]
